# Thermal and desalination performance enhancement of single slope solar still using phase change material

**DOI:** 10.1038/s41598-025-95894-y

**Published:** 2025-04-04

**Authors:** Suvanjan Bhattacharyya, Surjo Das, Sumit Khatri

**Affiliations:** Department of Mechanical Engineering, Birla Institute of Technology and Sciences Pilani, Pilani Campus, Vidya Vihar, Pilani, Rajasthan India

**Keywords:** Phase change material (PCM), Single slope solar still, Solar desalination, Fe_3_O_4_ nanoparticle

## Abstract

The study that is being presented focused on the numerical analysis of the melting regime for various phase change materials (PCMs) in order to select an optimal material that would enhance the desalination efficiency of single-slope solar stills. While choosing the PCMs, the following factors were considered, availability, economic viability, environmental friendliness, and thermophysical properties. The study utilised ANSYS Fluent 18.1 to conduct a comparative analysis based on the melting of five different PCMs at different time stamps. The models and results showed that at 5000 s, Fe_3_O_4_ nanoparticle-enhanced PCM is the most effective of all the PCMs that were studied. This is because it melted completely before the other PCMs, which included RT35, Lauric Acid, CaCl_2_·6H_2_O, and *n*-octadecane. The best inorganic PCM was discovered to be CaCl_2_·6H_2_O, which had a maximum liquid fraction of around 68%. The best organic PCM was determined to be *n*-octadecane, which had a liquid fraction of nearly 57%. Lauric acid and RT35 achieved maximum liquid fractions of approximately 49% and 41%, respectively.

## Introduction

Water is an integral part of sustaining our lives on Earth. It covers about 71% of our planet, out of which only 3% accounts for freshwater and just 1.2% is safe for drinking purposes. With urbanization proliferating, access to clean drinking water has become a major concern. The depletion of fresh water resources is mainly caused by deforestation, population growth, and excessive consumption of groundwater. India, with its increasing population, now holds the title of the most populous country in the world and is also leading in terms of groundwater extraction. More than 1000 blocks in India have become water-stressed. To tackle this issue, the Indian government established entities such as the Ministry of Jal-Shakti in 2019 and the Central Groundwater Board (CGWB) to raise awareness and develop effective methods for conserving this valuable resource. To date, projects and initiatives such as groundwater level modeling, aquifer rejuvenation, and exploratory drilling have been implemented. A major problem with the ongoing projects is that we are not focusing on harnessing the full potential of the seawater. Desalination is a method of obtaining distilled water from seawater by removing the salt mineral present in it. This can be implemented as a viable solution. Very few countries such as Saudi Arabia and Israel have been able to utilize desalination as a method of having potable water. However current techniques of desalination are expensive and inefficient which is why countries abstain from using them. Our objective should be to find an effective way of desalination which would be both eco-friendly and cost-effective. Solar stills are currently being used for the process of distillation and desalination. The solar still is a transparent cage-like structure that facilitates a cycle of evaporation and condensation to purify water. To enhance the productivity of this process, various external means of heating are being studied and employed. In this context, the study and use of phase change materials (PCMs) is imperative. PCMs are unique materials with high latent heat of energy, which means that they either absorb or release tremendous amounts of energy during phase transition. This unique property of PCMs has garnered attention in the thermal energy industry as it can be applied in both heating and cooling processes. Another fascinating thing about PCMs is that they store energy during the day and release it in night. This promotes their longevity and adds to their practicality.

### Literature study

Moreno et al.^1^ worked on single-slope solar still and analyzed the effect of PCM in enhancing its productivity. They performed numerical simulations for different PCMs such as RT54 HC, RT62 HC, RT70 HC, and RT80 HC for different thicknesses of 1 cm, 0.5 cm, and 0.25 cm. Of all the tested cases, they concluded that PCM RT70 HC with a thickness of 0.25 cm yielded the best result. They carefully studied and interpreted the velocity, temperature, and water vapor mass fraction contours. They further observed that PCM showed maximum activity from 3 to 6 pm and the PCM. They agreed that the mass of the PCM taken plays a crucial role and that the use of PCM helped in increasing the efficiency of the solar still from 55.78% to 66.7%. Manoj Kumar et al.^2^ worked on a similar single slope solar still using a unique nano disbanded phase change material (NDPCM). Crude wax (CW) was used as organic PCM and lightweight Zinc Oxide (ZnO) nanoparticle was used. They performed a comparative analysis between a normal still, CW still and a still with the NDPCM by comparing the daily fresh water output for each case. Upon addition of one percent of ZnO nanoparticles in the organic PCM, it was observed that the efficiency of the solar still enhanced from 50.24% to 65.17%. The increased efficiency was due to an increase in the thermal conductivity of crude wax upon addition of ZnO particle which was greater than its base value by 27.78%. Stepped solar still basins are also a popular modification to the conventional solar stills used as the efficiency is generally higher. Hussain et al.^3^ worked on a stepped solar still with the basin filled with Lauric acid as PCM. The stepped solar still consisted of four steps inclined at an angle of 30 degrees to the ground. Saline water levels of a minimum of 10 mm and a maximum of 40 mm height subjected to constant boundary conditions were analyzed. The PCM phase transition was studied using the enthalpy porosity or solidification and melting model on a finite element-based CFD model package. The various contours which were studied by the authors during the simulation of the 2D model included enthalpy, velocity, pressure, mass fraction and temperature. Interestingly, the temperature distribution was not uniform and was majorly affected by the inclination provided in the setup. The PCM melted partially, only 50% yet the minimum temperature difference recorded was 1.2 °C and the maximum temperature difference recorded was 4 °C. Gugulothu et al.^4^ experimentally verified the effect of different inorganic PCMs in enhancing the productivity of a solar still. They used a single slope solar still having a 100 × 120 base, vertical and lateral height of 100 and 146 respectively and an inclination angle of 32 degrees to the base. The use of potassium dichromate (K_2_Cr_2_O_7_), magnesium sulfate heptahydrate (MgSO_4_.7H_2_O) and sodium acetate (CH_3_COONa) was done as PCMs. The comparison was drawn on the basis of temperature variation with time while operating the solar still with and without the PCMs. The key factor the authors noted in this experimental validation was that the incident solar radiation played a crucial role. The incident radiation was hence measured for all configurations using a pyronamometer. Of all the tested PCMs, MgSO_4_.7H_2_O yielded the best results making it a suitable PCM for obtaining potable water from this setup. Khandagre et al.^5^ worked on improving the performance of solar using MgSO_4_.7H_2_O as the base PCM. In order to draw a good comparative analysis in terms of productivity, a double-slope solar still with a basin area of 0.5 × 0.5 m^2^ was used which can be thought of as a combination of two single-slope solar stills. Three sets of observations were taken with difference in the amount of PCM used in the solar still. The authors studied the water obtained at different time stamps and measured the corresponding intensities as well. Additionally, it was concluded that the specific heat energy holds an inverse relation with the rate of heat addition and rejection and rate of evaporation hence the PCM is to be chosen wisely. The productivity of the solar stills was 1800, 1900 and 1960 ml/ m^2^/day when 0.5, 0.75 and 1 kg of MgSO_4_.7H_2_O were used respectively recorded as compared to 1400, 1420 and 1400 ml/m^2^/day of solar still without MgSO_4_.7H_2_O. The temperature difference increased by 80% when using PCM. The overall thermal efficiency was found to be 47% without PCM and 67% with PCM. Al-Hamadani et al.^6^ used a similar double slope solar still with a base area of 1 m^2^ and an angle of inclination of 65 degrees to test the efficiency of the solar still using organic Myrisitic acid of 99.2% purity. To simulate the heating effect due to the sun an external heating source in the form of electrical energy was used. For the given setup, using 20 kg of Myrisitic acid gave the maximum productivity. The weight of the PCM to be added is something that cannot be overlooked this is because after a certain amount the mass of PCM used, holds an inverse relation with the heating. This leads to non-uniform distribution of heating across the solar still which makes it ineffective. The use of Myrisitic acid drastically increased the productivity of the solar still by 35–40%. Maduabuchi et al.^7^ innovatively used the PCM *n*-octadecane in a solar thermoelectric generator (STEG). They placed it at the hot junction of the STEG to decrease the adverse effect of transient and non-uniform solar radiation on the generator. The use of PCM in the STEG helped in minimising energy fluctuation, loss of radiation energy and increasing electrical stability. The PCM was primarily used in the liquid state hence Navier Stokes was used to study the liquid PCM behaviour. Simulations of the PCM at the hot junction of the STEG were done on ANSYS 2020 R1 software and further validation was using Matlab. Abdullah, et al.^8^ worked on a single slope solar still embedded with a layer of paraffin wax PCM and Copper Oxide (CuO) nanoparticle. The set-up was modified by using internal collectors which improved the efficiency. The use of internal reflectors and nano-coating resulted in the trays distiller having total freshwater more than the traditional distiller by around 70.7%, where the yield records 4100 and 2400 ml m^2^ their study, Seifi et al.^9^ investigated the thermo-economic performance of a parabolic solar collector that was integrated with PCM in the receiver tube for solar desalination. They examined the function of the PCM in energy storage, evaluated the thermal efficiency of the system, and assessed its economic viability. The model illustrated improved heat retention and enhanced desalination efficiency, demonstrating its economic feasibility. Santhosh et al.^10^ worked on a single-slope solar still where they used Paraffin wax infused with iron oxide particles as PCM. To increase the efficiency further, a solar water heater was connected externally to set up. The efficiency increased from 20.08% for a normal solar still to 25.11% for an enhanced solar still. Furthermore, the solar heater had an improvement of 25% over enhanced solar still. The heat stored by PCM was used for operating the solar still even after sunset. RAJU et al.^11^ went a step further and used extended surfaces to facilitate heat transfer. A comparative analysis between three types of single slope solar stills was done, a simple one, another with solar still having fins over baseline and a finned solar still induced with PCM. Cylindrical copper tubes were arranged in a linear pattern. Paraffin wax was used as PCM. The single slope solar still, the finned and fin induced PCM solar still yielded 1670 ml water per day, 1990 ml water per day and 2530 ml water per day. Thus, on comparing efficiencies for the single slope solar still to finned and fin-induced PCM solar still, it was found that the latter was more productive that too by a margin of 17.54% and 55.69% respectively. In a similar experiment, Manoj Kumar et al.^12^ conducted an experimental analysis on three arrangements of a single slope solar still. Firstly, single slope solar still. Secondly, they used the solar still embedded with inorganic PCM which was sodium acetate trihydrate (CH_3_COONa.3H_2_O) salt. Lastly, the inorganic PCM solar still was combined with an NPCM in the form of magnesium oxide nanoparticles. The daily water collection was found to be 2520 ml and 2890 ml for the solar still embedded with inorganic PCM and inorganic doped NPCM solar still respectively, which is 26.63% and 45.23% higher than the simple single slope solar still, respectively. Interestingly, in the study an appreciable temperature rise was observed in the modified solar still setups. Dhivagar et al.^13^ did an experimental as well as numerical analysis to compare the performance of a single slope solar still and a gravel coarse aggregate assisted solar still. They used the ANSYS FLUENT 19.2 software for this purpose. They too found an appreciable in the temperatures in the two stills. The distillate of the gravel coarse aggregate assisted solar still was greater than the single slope solar still by a fraction of 23.3%. El-Sebaey et al.^14^ worked on a similar single-slope solar still in an Egyptian climate. Their efforts helped in showing how to optimize the output of a single-slope solar still in any geographical location and condition. The daily simulated and experimental productivity was found to be 1.982 and 1.785 l/m respectively with a water depth of 2 cm. The daily simulated and experimental efficiencies were 16.79% and 15.5%, respectively. Kabeel et al.^15^ in a theoretical study compared the performance of multiple PCMs in a single-slope solar still. They took into account various thermophysical properties, economical feasibility and environmental friendliness for the selection of the ideal PCM. Capric-palmatic PCM was the best when it came to economic feasibility and environmental impact however, PCM A48 stood out in terms of performance and also because of its environmental friendliness as an organic PCM. Omara et al.^16^ reviewed different experimental analyses on solar stills of different orientations and shapes along with the suitable PCMs used in each setup. Their review study indicated productivity improvement of up to 120% of a PCM-embedded solar still as compared to a solar still without PCM. They further found that the productivity of an active solar still with PCM could reach as high as 700%. They further could conclude that because of rising environmental concerns and taking into account economic feasibility, researchers preferred using organic PCMs such as Paraffin wax. Ketut et al.^17^ in an experimental study tried to validate if Myrisitc acid could be used as a possible PCM in an Indonesian climate. While the results were not successful in the day as the PCM could not attain its melting temperature of 58 °C for the ambient temperature was 42.5 °C however, during the night it was observed that there was some improvement in the distillate produced by the double slope solar still setup. Li et al.^18^ did an experimental and theoretical analysis of a unique spherical thermal storage device filled with a composite phase change material comprising myristic acid and expanded graphite. The effects of the manipulation of Stefan number and the extended graphite content were analyzed. It was found that as the extended graphite content was increased, the PCM melting was also enhanced. When extended graphite contents were 4 wt.%, 5 wt.%, and 6 wt.%, the time required to complete melting in the unit was reduced by 82.2%, 85.6%, and 88.0%. The Stefan number however held an inverse relation with the melting of the PCM unit. When the Stefan number was decreased from 3.43 × 10^−1^ to 9.50 × 10^−2^, the complete melting time of the material in the unit was increased by 58.9%. The innovative idea of using extended graphite and finding correlations between non-dimensional numbers made this study quite interesting. Faraj et al.^19^ did a comprehensive study on the different types of PCMs being used in different thermal energy storage devices. Their focus was on using PCM for cooling purposes in buildings. Although the presented paper is more of a review of previously done work however it gives a good insight into the various classifications and uses of PCMs. The classifications and findings were presented were presented very meticulously which makes the selection of a suitable PCM easier. Khairat Dawood et al.^20^ did an experimental analysis on a single-slope solar still. The interesting thing to note in this work is that the use of CuO nanofluids was done and also an external vibration was induced to enhance the productivity. To support the results, the authors also did an effective cost analysis which further went to show how efficient the process is. The solar still setup with vibration and CuO concentration of 1.5% at a water depth of 10 mm yielded 7.13 kg of water which was by far the best result. Dawood et al.^21^ in the presented work investigated ways to improve the productivity of solar stills by combining an electric heater, vibration motion, and thermoelectric cooling to increase evaporation and condensation rates. The study, which took place in Alexandria, Egypt, demonstrated that this combined approach led to a significant increase in freshwater output, efficiency, and CO2 mitigation compared to traditional solar still systems. The maximum daily freshwater production using solar heaters, thermoelectric coolers, and vibration motion was 12.82 kg/day, with an estimated maximum cost of 0.01786 $/l/m^2^. Pan et al.^22^ conducted a study in which they explored unconstrained melting in a vertical cylinder, emphasising mushy region effects. The authors utilized experimental setups and numerical setups to examine phase change dynamics. The PCM used for the analysis was CaCl_2_·6H_2_O. The authors also established a linear correlation between the temperature difference created and the tuning parameter after validating it both experimentally and numerically. Chelliah et al.^23^ investigated the thermal performance and cost-saving potential of terracotta brick buildings integrated with PCMs. The study specifically uses paraffin-based PCMs to analyze temperature regulation and energy efficiency, demonstrating that PCM-enhanced bricks can significantly reduce indoor temperature fluctuations and improve energy savings in buildings. Five different PCMs were analyzed during this study namely OM18, OM29, OM32, and OM37 which are organic mixtures and HS22 which is a hydrated salt. The PCMs were tested for three sets of brick configurations with different numbers of columns (one, two and three). The arrangement of terracotta bricks in three columns filled with OM32 results in the highest annual air conditioning cost savings of $74.70 equivalent to 6281.01 INR and $61.90 equivalent to 5204.74 INR in hot-dry and composite climates in India, respectively. Hedwig et al.^24^ conducted a theoretical analysis in Germany in this paper where they presented a thermal optimization study of sorption processes using PCMs through a grad–div stabilized discontinuous Galerkin method. The study investigated how incorporating PCMs can improve heat and mass transfer efficiency in sorption systems. By using numerical simulations and optimization techniques, the researchers demonstrated the significant potential for optimizing thermal performance through strategic PCM integration. Embedding of PCM RT28HC increases the efficiency by about 13–50%. Wong-Pinto et al.^25^ in their paper reviewed advancements in incorporating nanoparticles into salt hydrates to improve their efficiency as PCMs. The addition of nanoparticles like aluminum oxide (Al2O3), copper oxide (CuO), and carbon nanotubes (CNTs) enhances the thermal conductivity and stability of salt hydrates, addressing limitations like low thermal conductivity and subcooling. This leads to improved heat transfer performance, making them more effective for energy storage solutions. The authors concluded that the addition of nanoparticles enhanced the thermal conductivity of the salt hydrates and also that the performance of inorganic PCMs was better than organic PCMs. Soodmand et al.^26^ conducted a theoretical study to examine the melting and solidification behaviour of polyethylene glycol 1500 as a PCM in rectangular, triangular, and cylindrical enclosures. Through these numerical simulations, they were able to analyze the heat transfer performance and phase transition efficiency across different geometries. The findings emphasize the influence of enclosure shape on the thermal performance of PCM, providing valuable insights for the optimization of energy storage systems. Ebadi et al.^27^ paper conducted an experimental and numerical study to analyze the melting of RT35 PCM inside a circular thermal energy storage system. They tried to manipulate various thermal and geometric parameters to study the influence. Their study helped to provide insight into optimising a circular thermal energy system embedded with PCM. L.M. Simeza et al.^28^ in their presented explore the influence of different geometric configurations on the thermal conduction shape factors of prismatic cylinders. They studied the heat transfer characteristics between isothermal boundaries and were also able to provide insights into optimizing thermal performance in engineering applications involving hollow prismatic structures.

### Novelty

The literature review reveals that there has been extensive numerical as well as experimental study conducted for understanding and improving the performance of a single slope solar still for desalination and it further reveals that the addition of PCMs undoubtedly, enhances the productivity of a solar still. The review also indicates that there are very few materials which have been explored that could be used as PCMs to increase the productivity of a solar still. Moreover, there are only a few PCM geometries that have been used for phase change analysis such as rectangular or circular. In our presented work, we are using a unique triangular geometry for studying the phase change analysis. Further, we are conducting numerical investigations on five lesser-known PCMs namely inorganic PCMs Rubitherm 35 and CaCl_2_·6H_2_O, organic PCMs Lauric Acid and *n*-octadecane, and a unique Fe_3_O_4_ nanoparticle incorporated in Rubitherm 35 PCM. This was the first time a numerical investigation was carried out on the Fe_3_O_4_ nanoparticle based PCM for a triangular geometry. The objective of the study is to find a suitable material with an optimum value of thermal conductivity and melting temperature, that can enhance the evaporation rate and overall productivity of a single slope solar still. Furthermore, the study also aims at understanding the influence of using an unconventional geometry on the melting regime of different PCMs.

## Computational methodology

**Fig. 1:**
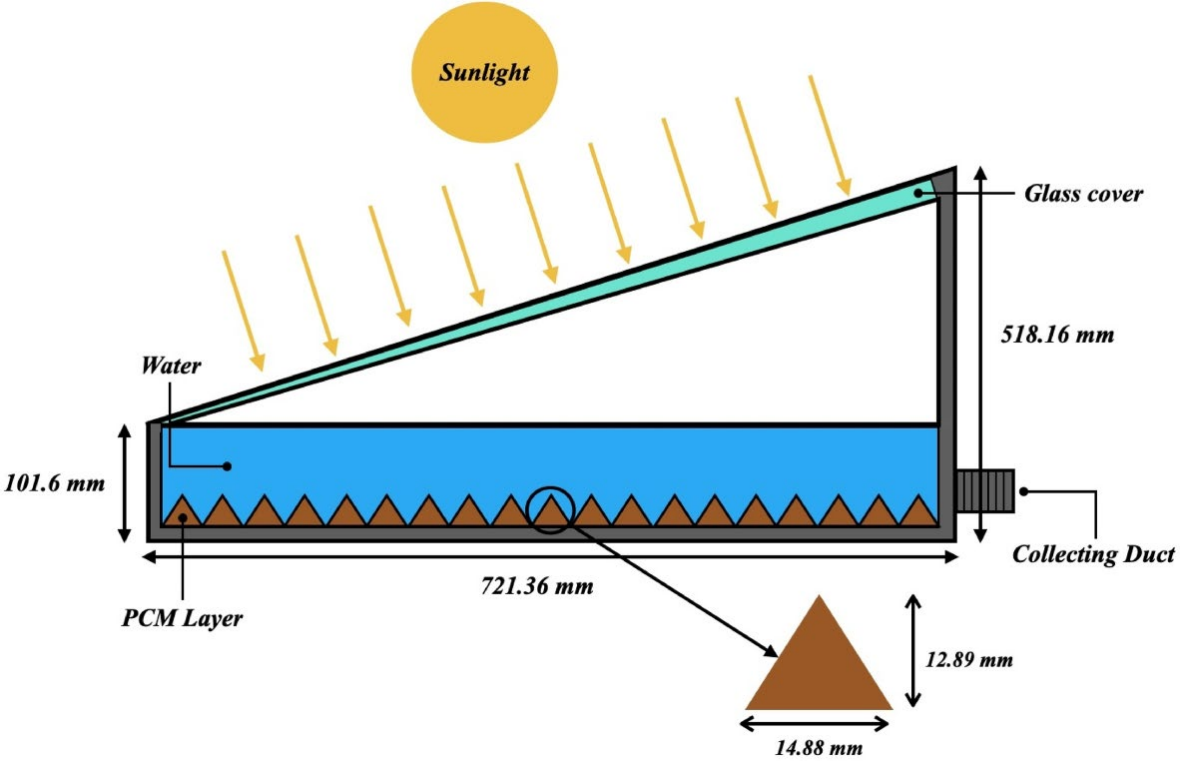
Proposed schematic of single slope solar with PCM basin.

The single slope solar still depicted in Fig. 1. uses a triangle geometry enclosed with PCMs, which was imported into ANSYS 18.0 Workbench for simulation purposes. Understanding how the melting process would take place from one of the faces of a pyramidal or conical geometry—which may be utilized in a real solar still—was the goal behind selecting the mentioned design. Soodmand's work served as the inspiration for the triangular geometry that was employed^26^. The base length of the geometry is 14.88 mm, and its height is 12.89 mm. This results in a total surface area of almost 96 mm^2^.

The melting analysis was done on ANSYS 18.0 Fluent software. A transient model was used in the software. The process was initialized by setting the initial temperature at 298K followed by gradual heating from all zones. The heating was done from only one side of the triangular model keeping the other two sides isothermal. A time step of 0.5 s was used, ensuring that there were twice as many time steps as there should have been for simulating melting. The time stamps at which the data was recorded were 100, 250, 500, 750, 1000, 1250, 1500, 2000, 2500, 3000, 3500, 4000, 4500 and 5000 s. The data collected included liquid fraction, temperature, wall shear stress and skin friction coefficient for the various time stamps. The resultant contours were obtained on CFD Post. In our numerical study, we conducted simulations following a similar approach to Soodmand et al.^26^. To simulate the melting process, heat was applied from one side of the PCM whereas the base and other end were assumed to be adiabatic. The entire wall of the PCM was initialized at a temperature of 298 K and heat was applied from all zones. Losses due to the convective mode of heat transfer were neglected. The model was made temperature-independent to make the analysis simpler. For the NPCM mixture of RT35 + Fe_3_O_4_ nanoparticles, a similar approach as Soodmand et al.^26^ was used because the mixture exhibits varying specific heat capacity, viscosity, and thermal conductivity in a piecewise linear manner with temperature. The Boussinesq approximation was used in this system to apply natural convection within the PCM. The QUICK spatial discretisation scheme was utilised to solve the momentum and energy equations. Additionally, the PRESTO scheme was chosen for the pressure correction equation, and the semi-implicit method for the pressure-linked equations (SIMPLE) algorithm was used for the pressure–velocity coupling.

## Governing equations

As assumed by Ebadi et al.^29^ in their study of experimentally and theoretically analyzing the melting of NPCM inside a thermal energy storage in the shape of a cylinder, in this paper we have used the same set of equations and have hence assumed the molten or liquid form of NPCM to behave like an incompressible Newtonian fluid and further considered laminar flow for the liquid phase of the NPCM. The density of the liquid phase of NPCM, which happened to vary linearly with temperature, had been modeled by adopting the Boussinesq approximation. It was also assumed that there was a local thermal equilibrium between the PCM and nanoparticles. An axisymmetric model was used to simplify the problem. As the melting process began, thermal energy flowed from the sides of the enclosure to the bio-based nano-PCM. As time passed, the liquid–solid interface became visible. The principal differential equations governing the melting process are listed below:

Mass conservation Eq. ^29^:

1 $$\partial u/\partial r+u/r+\partial w/\partial z=0$$

Energy conservation Eq. ^29^:

2 $$\frac{\partial T}{\partial t}+u\frac{\partial T}{\partial r}+w\frac{\partial T}{\partial z}=\frac{k}{\rho {C}_{p}}\left(\frac{1}{r}\frac{\partial }{\partial r}\left(r\frac{\partial T}{\partial r}\right)+\frac{{\partial }^{2}T}{\partial {z}^{2}}\right)$$

Momentum conservation equation (radial direction)^29^:

3 $$\rho \left(\frac{\partial u}{\partial t}+u\frac{\partial u}{\partial r}+w\frac{\partial u}{\partial z}\right)=-\frac{\partial p}{\partial r}+\mu \left(\frac{{\partial }^{2}u}{\partial {r}^{2}}+\frac{1}{r}\frac{\partial u}{\partial r}-\frac{u}{{r}^{2}}+\frac{{\partial }^{2}u}{\partial {z}^{2}}\right)+{A}_{m}\frac{(1-f{)}^{2}}{{f}^{3}+\epsilon }u$$

Momentum conservation equation (axial direction)^29^:

4 $$ \frac{\partial w}{{\partial t}} + u\frac{\partial w}{{\partial r}} + w\frac{\partial w}{{\partial z}} = - \frac{1}{\rho }\frac{\partial p}{{\partial z}} + \frac{\mu }{\rho }\left( {\frac{{\partial^{2} w}}{{\partial r^{2} }} + \frac{1}{r}\frac{\partial w}{{\partial r}} + \frac{{\partial^{2} w}}{{\partial z^{2} }}} \right) + g\beta \left( {T - T_{m} } \right) + A_{m} \frac{{(1 - f)^{2} }}{{f^{3} + \varepsilon }}w $$

Here* u* and *w* are the components of velocity in the radial and axial directions, respectively. *T, t, k, ρ* and *C*_*p*_ represent temperature, time, thermal conductivity, density, and specific heat at constant pressure, respectively. *μ*, *g*, *β*, and *T*_*m*_ represent viscosity, gravitational acceleration, coefficient of thermal expansion, and the melting temperature, respectively.

Mushy zone parameters are used in the momentum Eqs. (3) and (4) to account for the transition from solid to liquid phase for the PCM. In the addition terms *A*_*m*_ is the mushy zone parameter = 10^5^, *ε* = 10^−8^ is a small number to avoid division by zero and *f* is the melting region identifier which takes values between 0 and 1 where *f* = 0 indicates the PCM being in the solid state, *f* = 1 indicates the PCM being in the liquid state and any interim value of *f* indicates the PCM being in the transition state.

The thermophysical property relationships as given in Eqs. (5)–(13) below are approximately suitable for the NPCM used in this paper, where subscripts ‘*nl*’*,* ‘*ns*’*,* ‘*n*’*,* ‘*l*’*,* ‘*s*’ refer to properties of liquid NPCM, solid NPCM, nanoparticles, liquid PCM, and solid PCM, respectively.

Density of nano-PCM, liquid phase^29^:

5 $${\rho }_{nl}=(1-\phi ){\rho }_{l}+\phi {\rho }_{n}$$

Density of nano-PCM, solid phase^29^:

6 $${\rho }_{ns}=(1-\phi ){\rho }_{s}+\phi {\rho }_{n}$$

Dynamic viscosity of nano-PCM, liquid phase^29^:

7 $${\mu }_{nl}=\frac{{\mu }_{l}}{(1-\phi {)}^{2.5}}$$

Thermal conductivity of nano-PCM, liquid phase^29^:

8 $${k}_{nl}=\frac{{k}_{n}+2{k}_{l}-2\phi ({k}_{l}-{k}_{n}){k}_{l}}{{k}_{n}+2{k}_{l}+\phi ({k}_{l}-{k}_{n})}$$

Thermal conductivity of nano-PCM, solid phase^29^:

9 $${k}_{ns}=\frac{{k}_{n}+2{k}_{s}-2\phi ({k}_{s}-{k}_{n}){k}_{s}}{{k}_{n}+2{k}_{s}+\phi ({k}_{s}-{k}_{n})}$$

Expansion coefficient of nano-PCM, liquid phase^29^:

10 $$(\rho \beta {)}_{nl}=(1-\phi )(\rho \beta {)}_{l}+\phi (\rho \beta {)}_{n}$$

Expansion coefficient of nano-PCM, solid phase^29^:

11 $$(\rho \beta {)}_{ns}=(1-\phi )(\rho \beta {)}_{s}+\phi (\rho \beta {)}_{n}$$

Heat capacity of nano-PCM, liquid phase^29^:

12 $$(\rho {C}_{p}{)}_{nl}=(1-\phi )(\rho {C}_{p}{)}_{l}+\phi (\rho {C}_{p}{)}_{n}$$

Heat capacity of nano-PCM, solid phase^29^:

13 $$(\rho {C}_{p}{)}_{ns}=(1-\phi )(\rho {C}_{p}{)}_{s}+\phi (\rho {C}_{p}{)}_{n}$$

## Grid independence test and validation

**Fig. 2:**
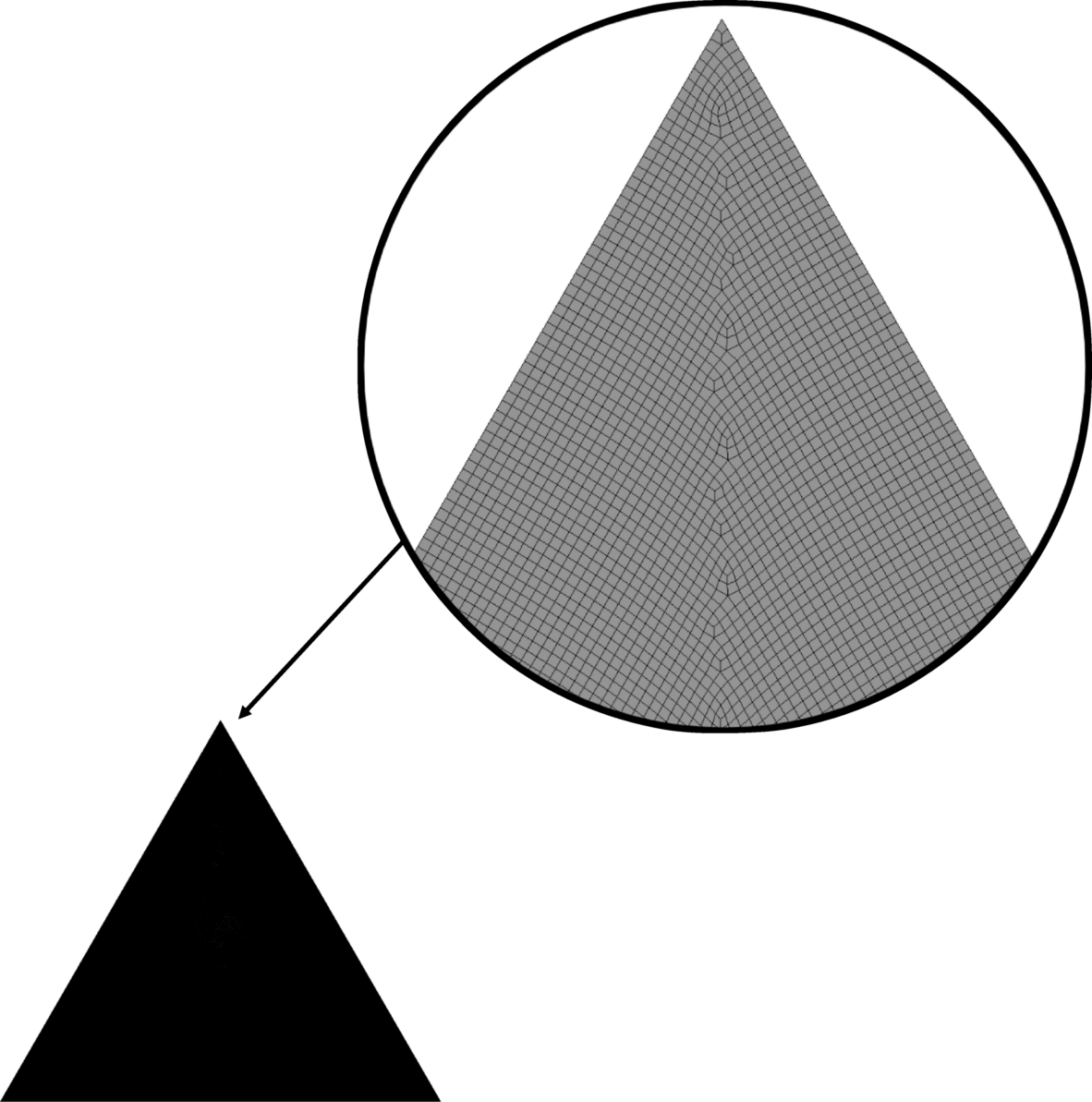
Meshing produced for numerical analysis of selected triangular geometry for mesh size of 0.1 mm.

**Fig. 3:**
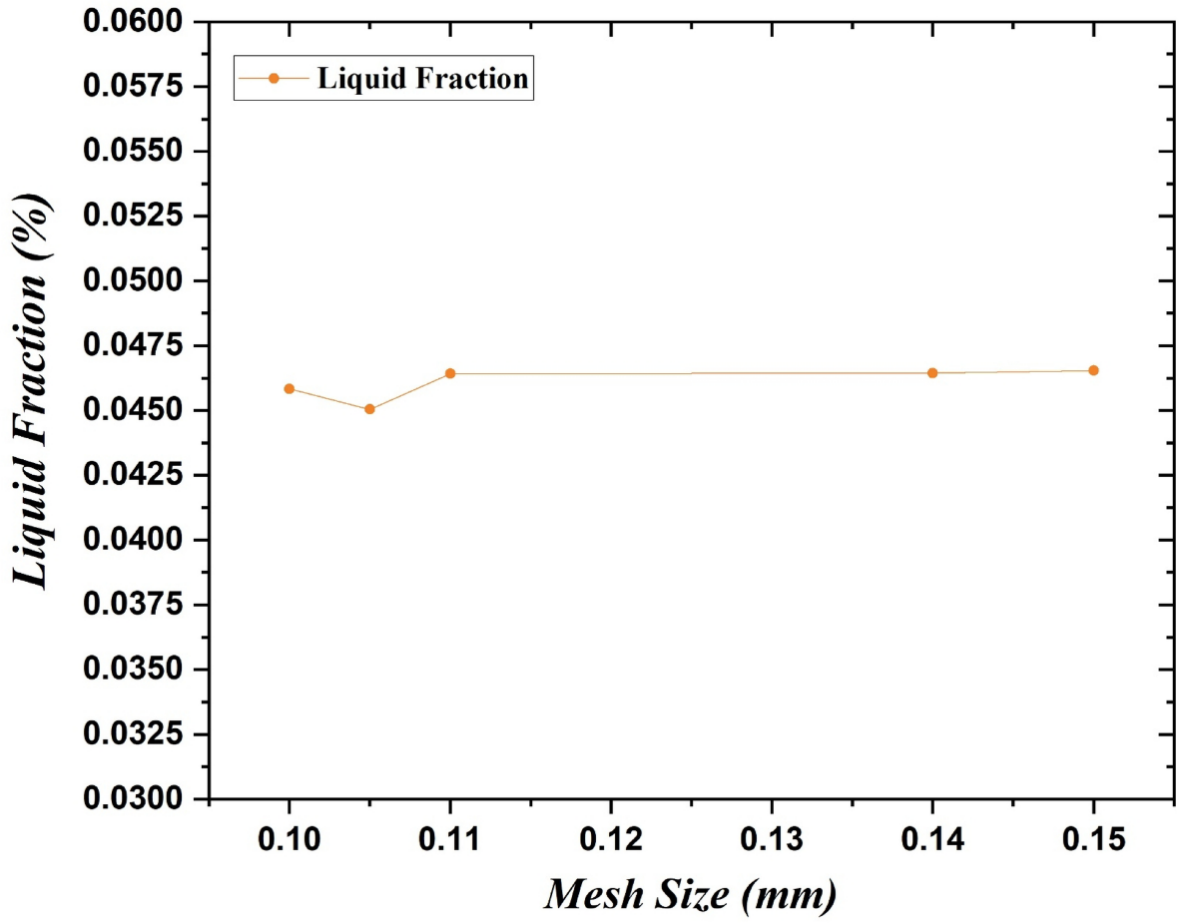
Grid independence test conducted on RT35 for mesh sizes of 0.1, 0.105, 0.11, 0.14 and 0.15 mm.

To obtain accurate results from our study, simulations were run on ANSYS Fluent 18.0 for the selected triangular geometry for a fixed time interval with different mesh sizes as shown in Fig. 2. The mesh chosen sizes were 0.1 mm, 0.105 mm, 0.11 mm, 0.14 mm and 0.15 mm and the number of nodes analyzed corresponding to the selected mesh sizes were 954,271, 887,918, 791,284, 505,750 and 442,595 nodes respectively. The timestamp selected for the comparison was of 100 s. All the simulations were run for the PCM Rubitherm 35. From the obtained test results as shown in Fig. 3, the relative difference in liquid fraction was close to 1% thereby declaring the independence of the mesh. Since there was not much variation in the output and a greater number of nodes would give more accurate results, for all our further simulations we had hence selected the mesh size of 0.1 mm.

**Fig. 4:**
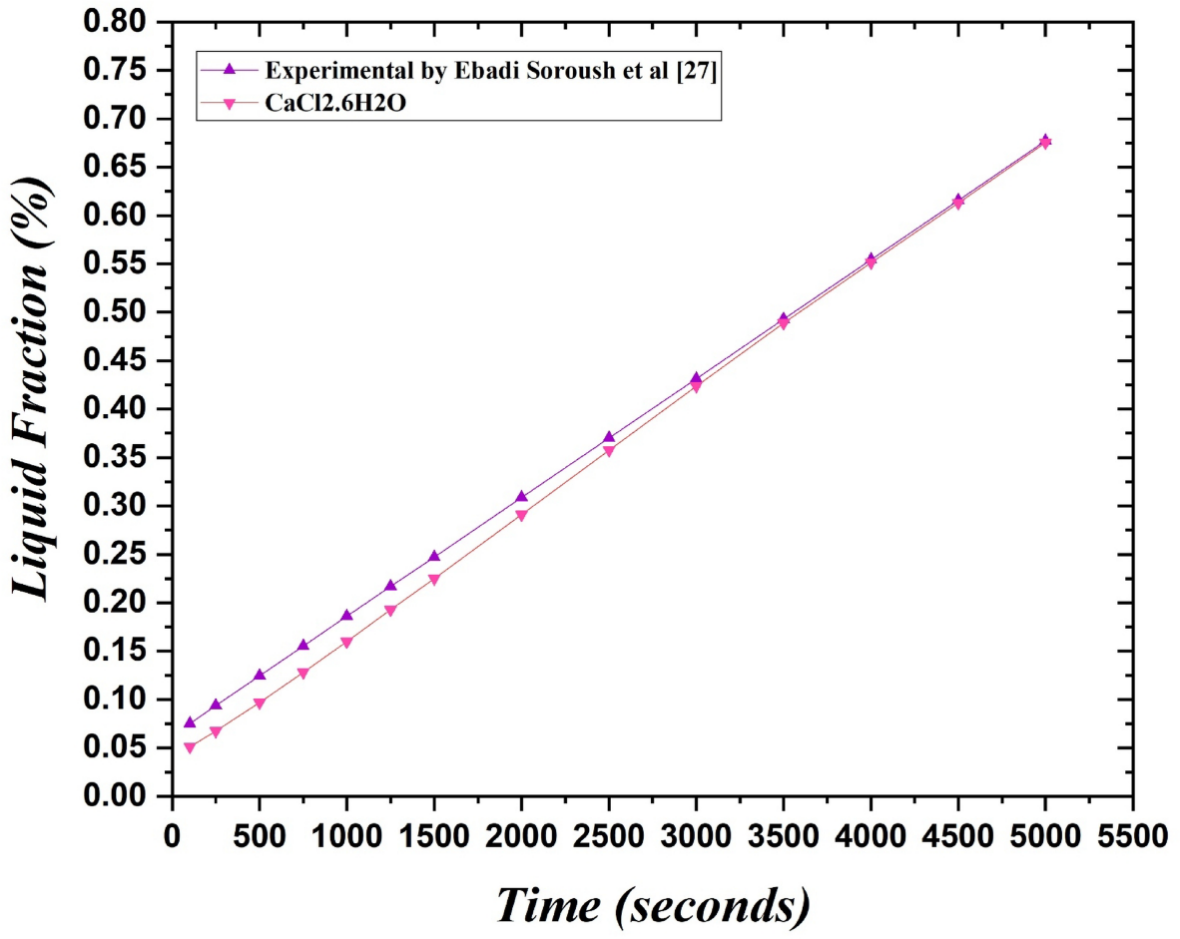
Validation of obtained results from numerical study of PCM melting with study conducted by Ebadi et al.^27^.

In order to verify our findings, we conducted a comparative analysis, as illustrated in Fig. 4, between our results and the numerical work conducted by Ebadi et al.^27^, wherein they had optimized a circular thermal energy storage system using RT35. Ebadi et al.^27^ had employed 33,905 nodes for their simulation, which was fewer than the number of nodes we had used for our simulation which was 954,271. Additionally, Ebadi et al.^27^ had used heat from the cylinders' lateral sides in their boundary conditions, which undoubtedly had a greater effect on speeding up the melting process. They results from our study validate our findings because they demonstrate a comparable pattern to Ebadi et al.^27^ with an acceptable relative error of 1%, despite having a smaller mesh size and heat conduction from a single surface.

## Results and discussion

The results obtained by utilizing different PCM for the given shape might be explained by the effect of varied latent heat of fusions, melting temperature and thermal conductivities. If the PCM has a lower melting temperature, it means that a smaller temperature difference is needed for the phase shift to occur. However, an exceptionally low melting temperature PCM may not persist in the solid state in normal room temperature settings so the melting temperature plays a critical role.

The PCM absorbs and dissipates more energy for phase shift when the latent heat of fusion is higher. All of the selected PCMs Lauric Acid, *n*-octadecane, CaCl_2_·6H_2_O, and RT35 have a constant thermal conductivity, with the exception of nano phase change material (NPCM). NPCM has a thermal conductivity that changes linearly, which makes it easier for heat to be transferred.

**Fig. 5:**
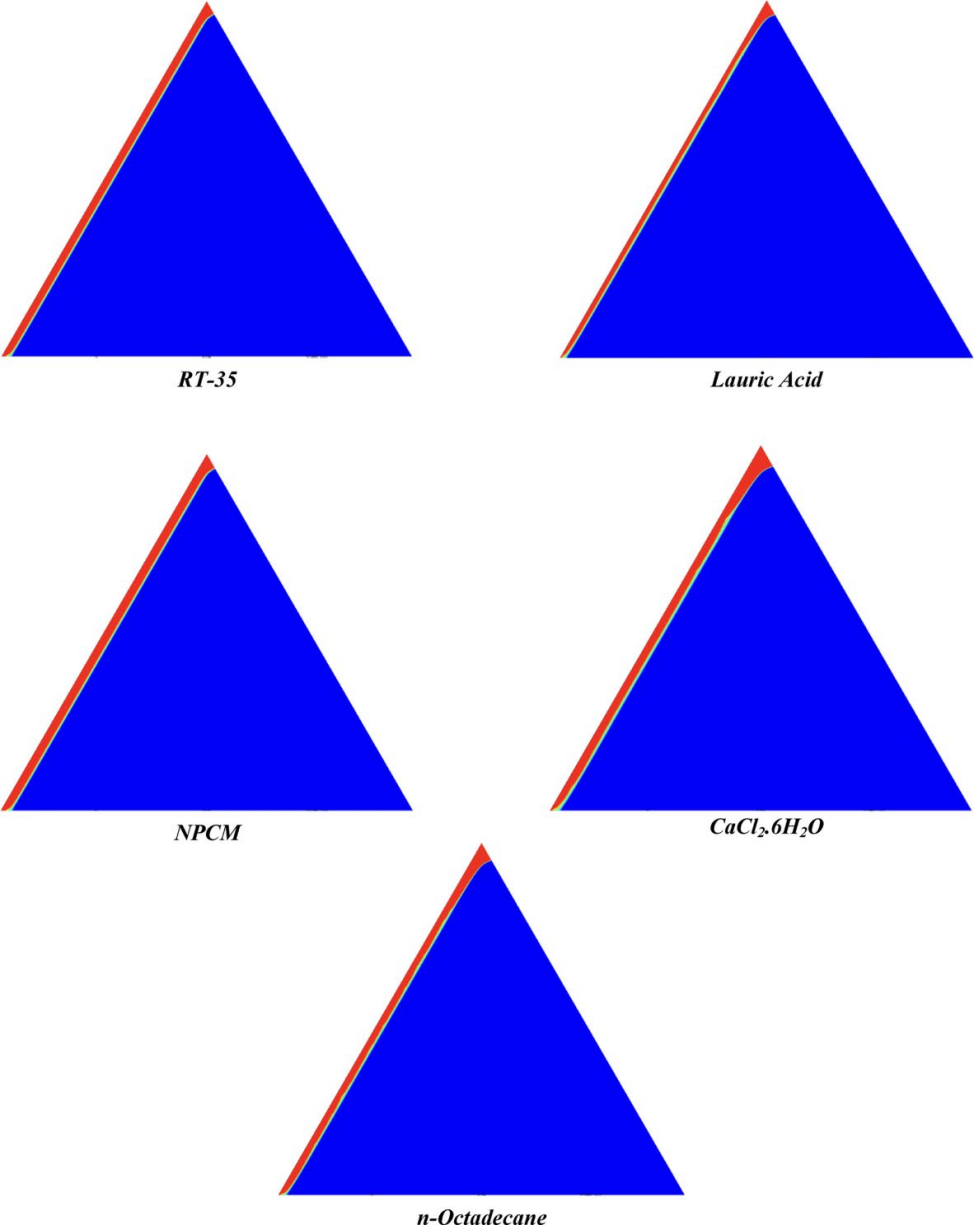
Melting regime of PCMs RT-35, Lauric Acid, NPCM, CaCl_2_·6H_2_O and *n*-octadecane at 100s timestamp.

**Fig. 6:**
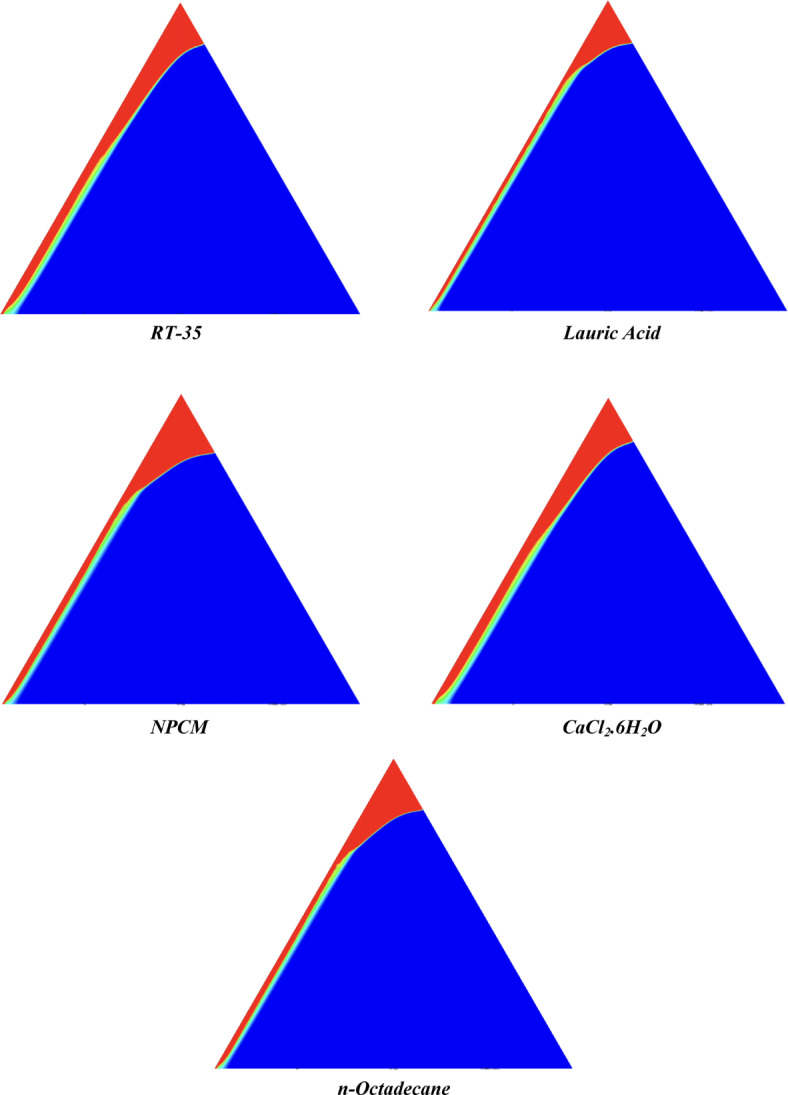
Melting regime of PCMs RT-35, Lauric Acid, NPCM, CaCl_2_·6H_2_O and *n*-octadecane at 250s timestamp.

**Fig. 7:**
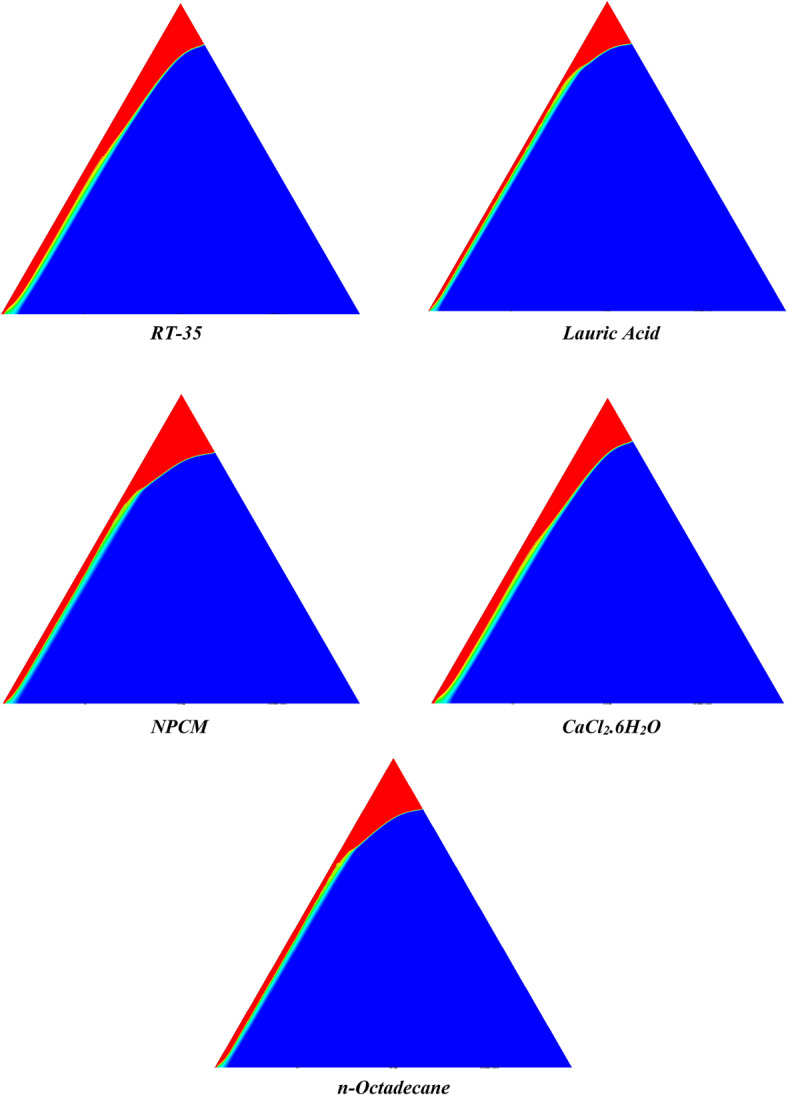
Melting regime of PCMs RT-35, Lauric Acid, NPCM, CaCl_2_·6H_2_O and *n*-octadecane at 500s timestamp.

**Fig. 8:**
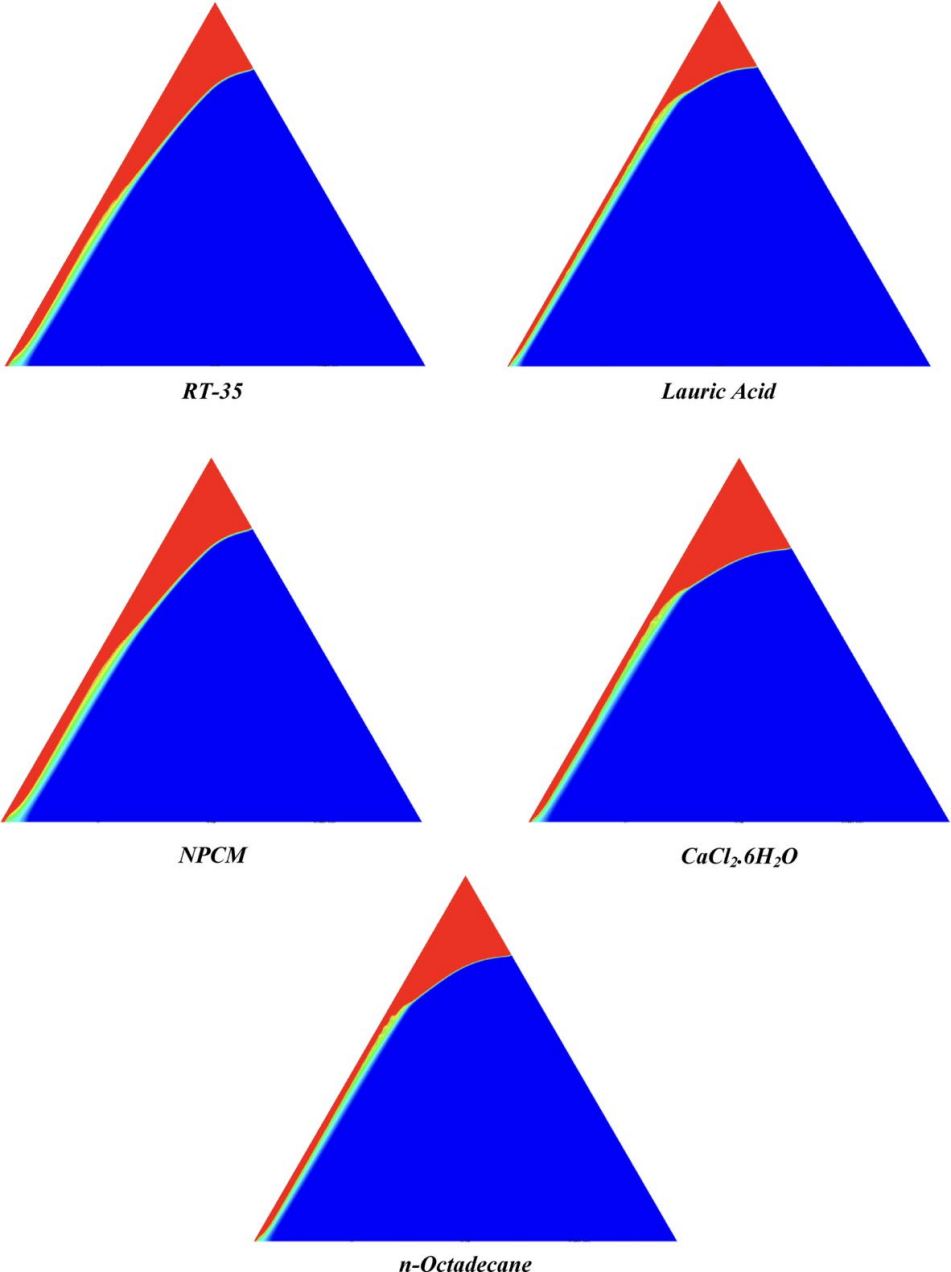
Melting regime of PCMs RT-35, Lauric Acid, NPCM, CaCl_2_·6H_2_O and *n*-octadecane at 750s timestamp.

**Fig. 9:**
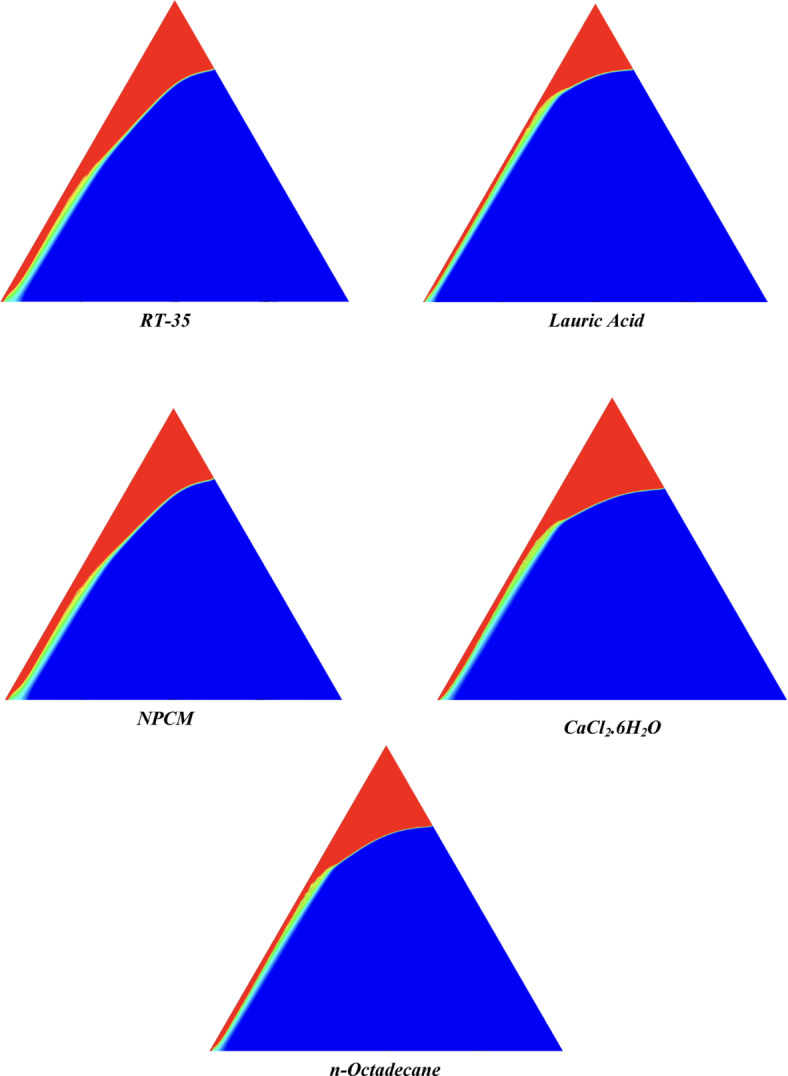
Melting regime of PCMs RT-35, Lauric Acid, NPCM, CaCl_2_·6H_2_O and *n*-octadecane at 1000s timestamp.

**Fig. 10:**
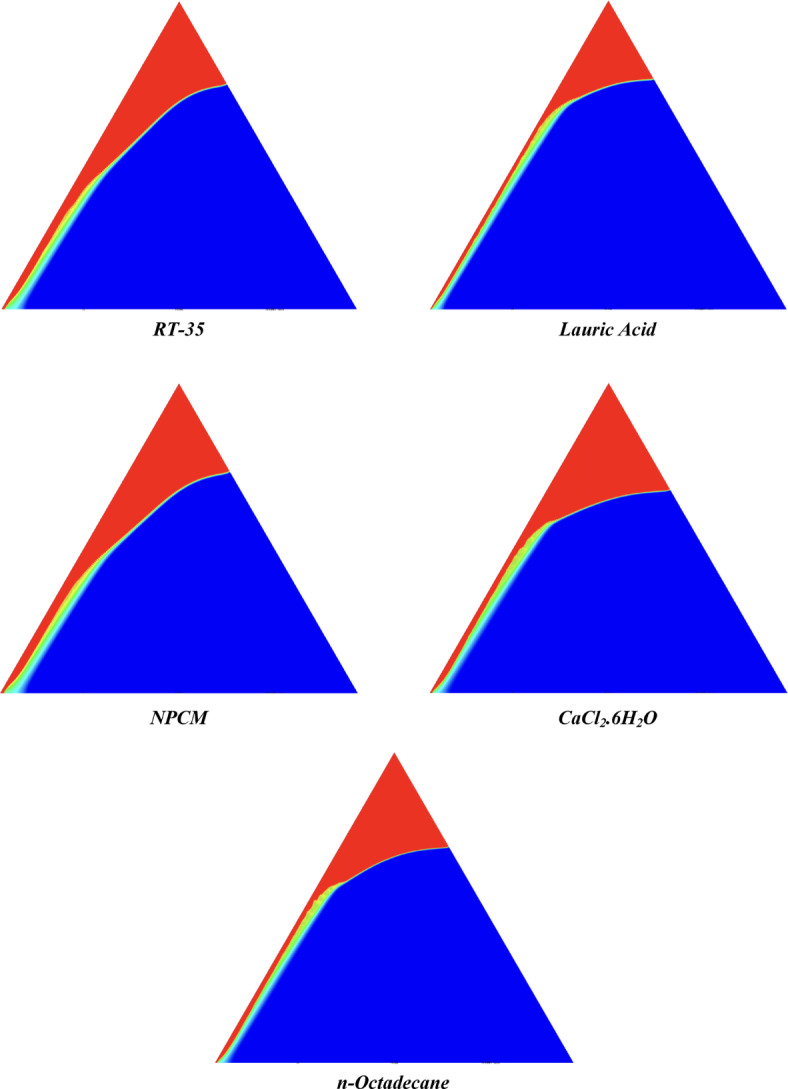
Melting regime of PCMs RT-35, Lauric Acid, NPCM, CaCl_2_·6H_2_O and *n*-octadecane at 1250s timestamp.

**Fig. 11:**
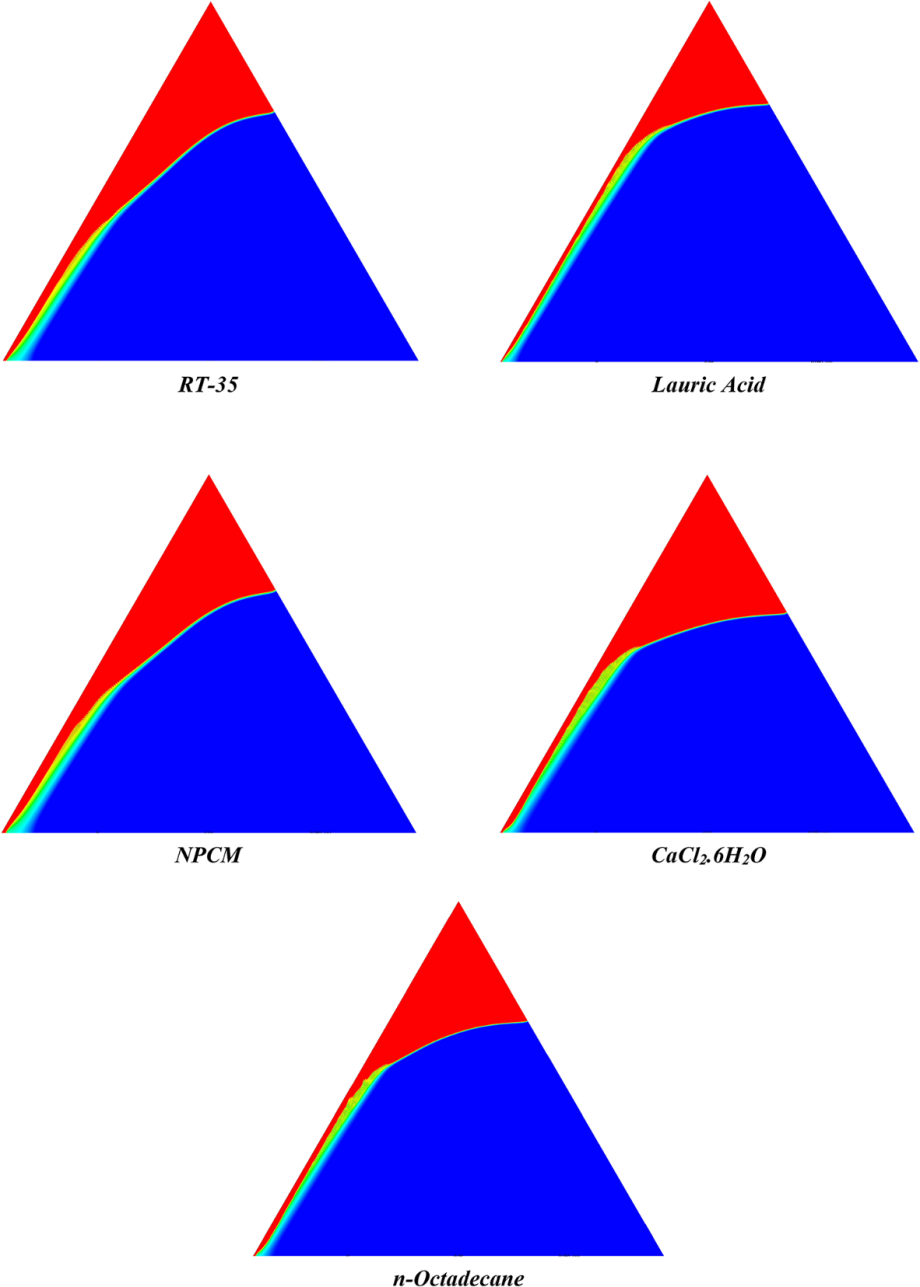
Melting regime of PCMs RT-35, Lauric Acid, NPCM, CaCl_2_·6H_2_O and *n*-octadecane at 1500s timestamp.

**Fig. 12:**
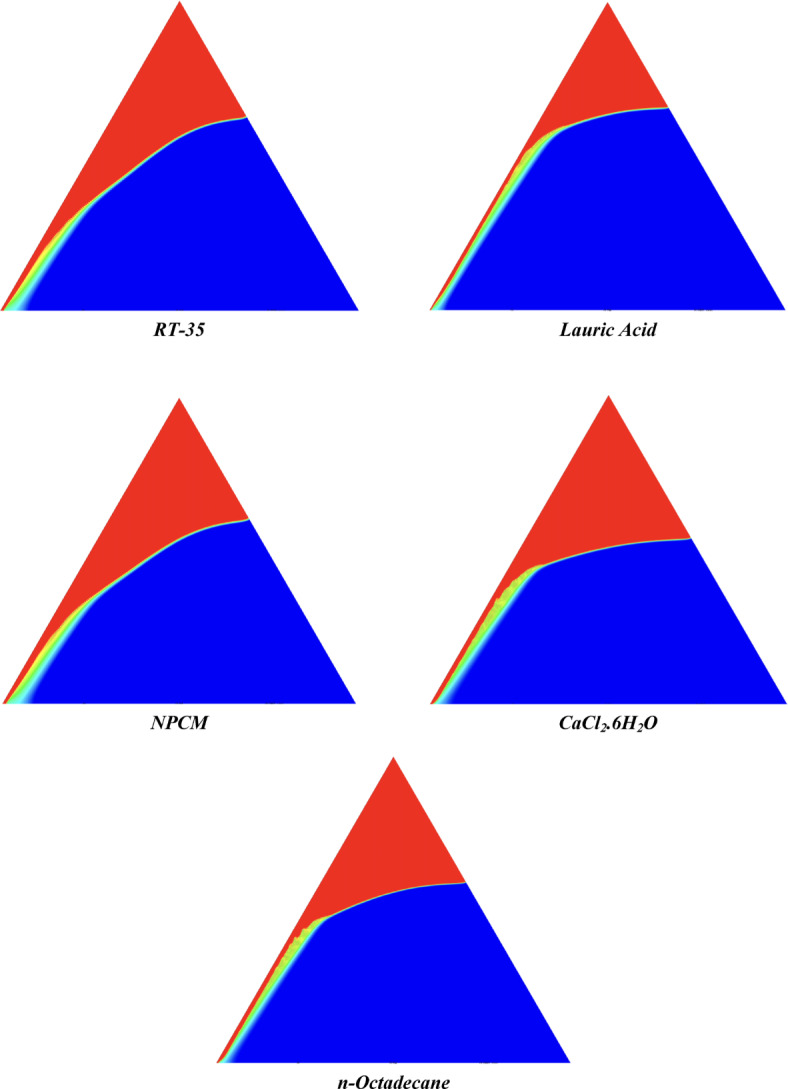
Melting regime of PCMs RT-35, Lauric Acid, NPCM, CaCl_2_·6H_2_O and *n*-octadecane at 2000s timestamp.

**Fig. 13:**
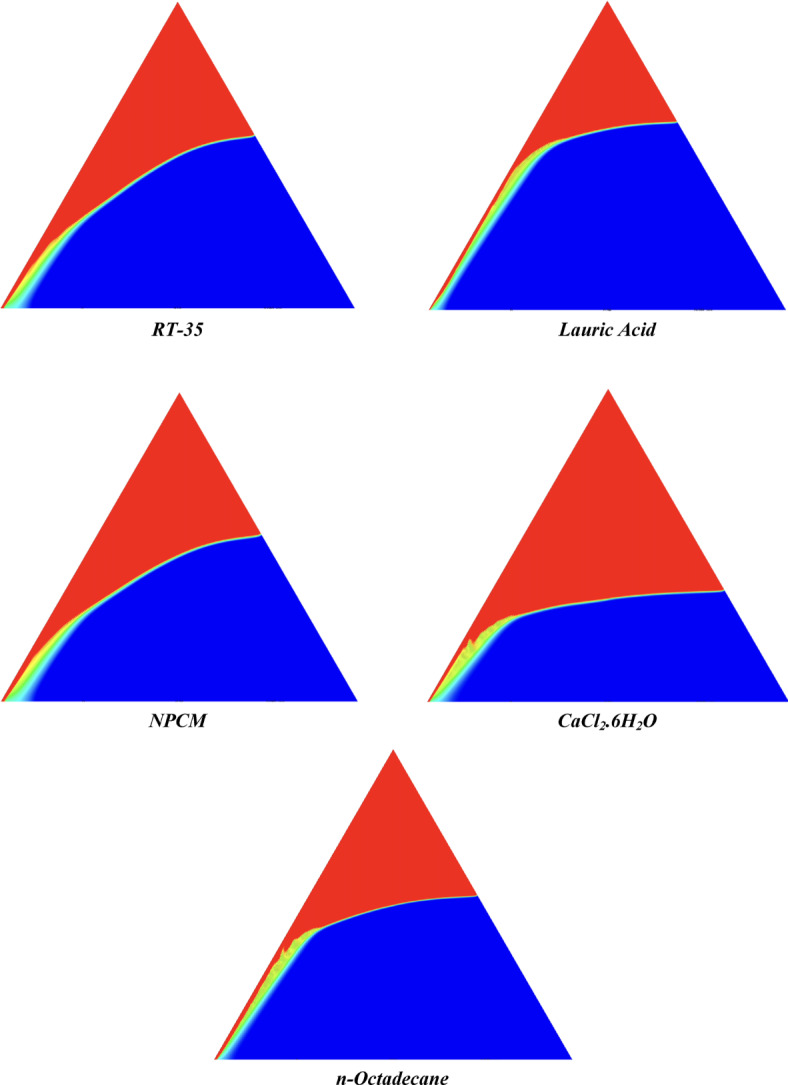
Melting regime of PCMs RT-35, Lauric Acid, NPCM, CaCl_2_·6H_2_O and *n*-octadecane at 2500s timestamp.

**Fig. 14:**
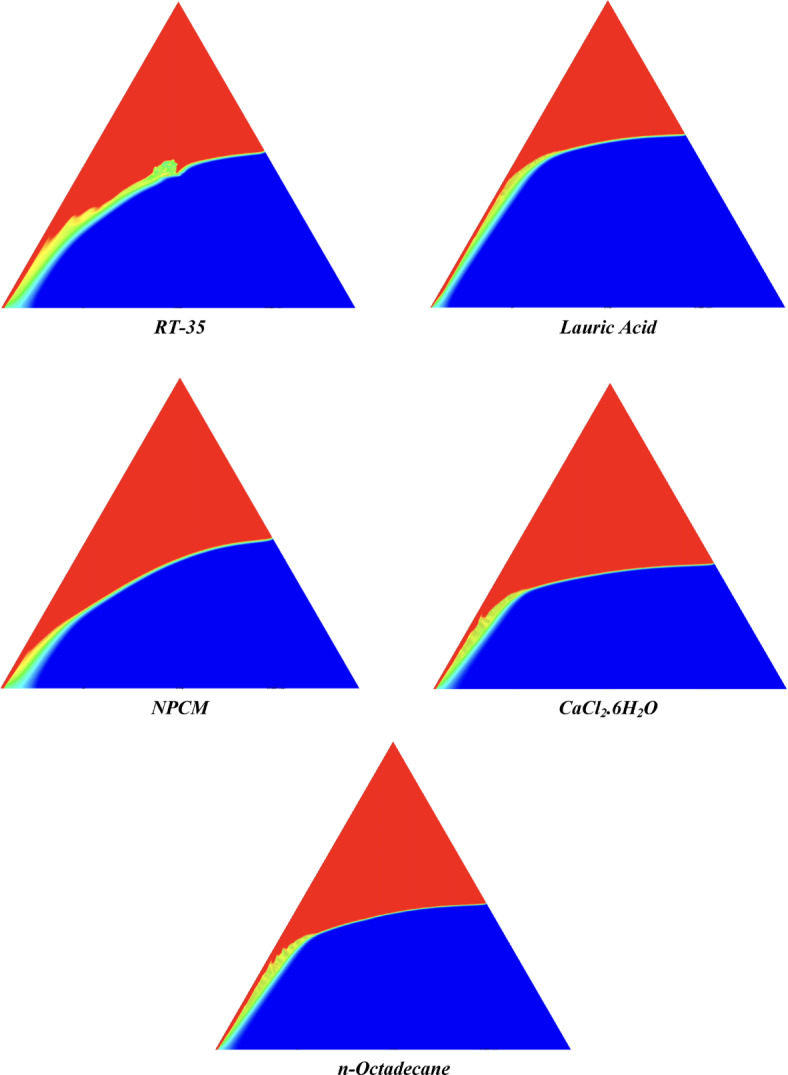
Melting regime of PCMs RT-35, Lauric Acid, NPCM, CaCl_2_·6H_2_O and *n*-octadecane at 3000s timestamp.

**Fig. 15:**
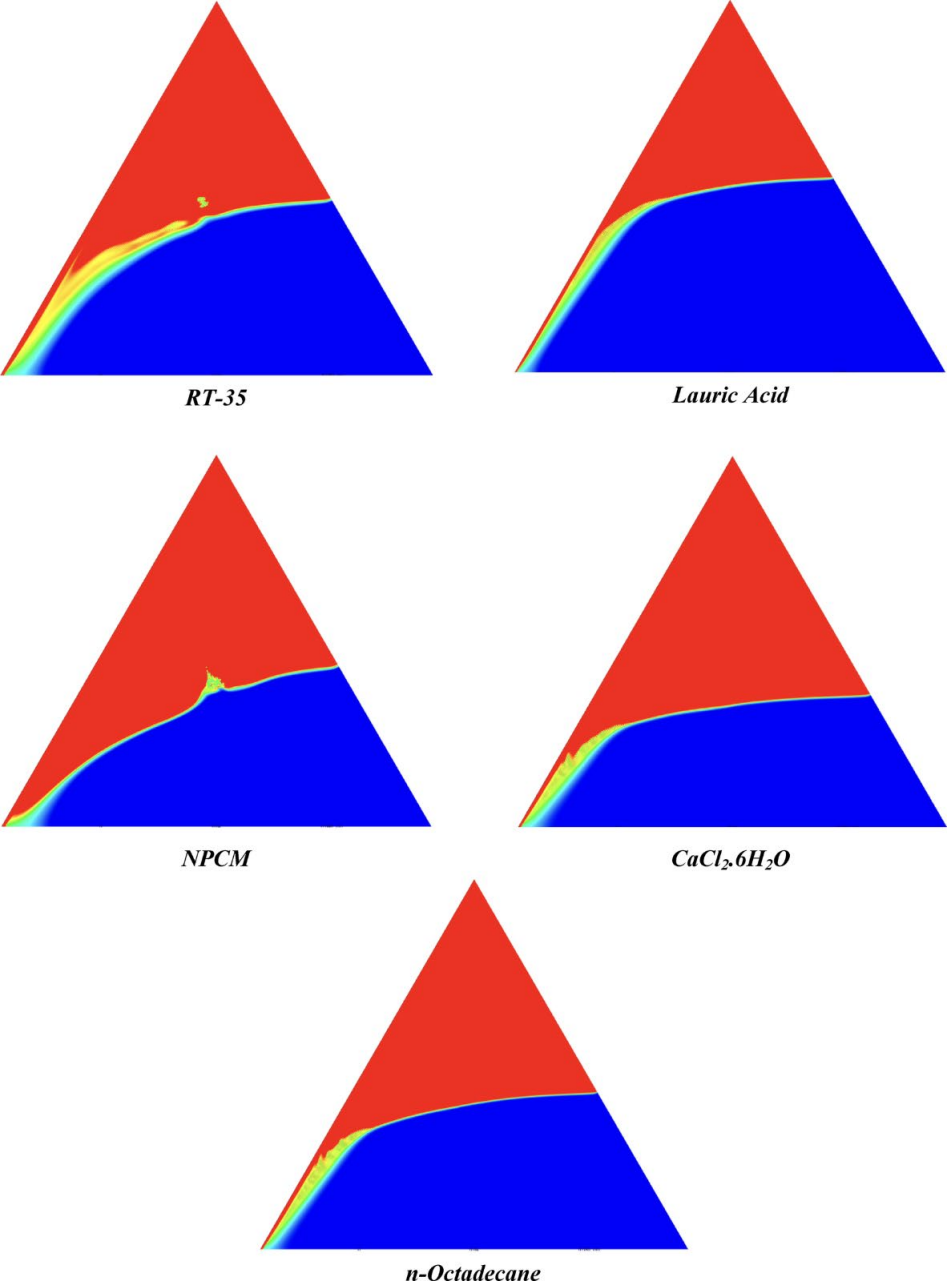
Melting regime of PCMs RT-35, Lauric Acid, NPCM, CaCl_2_·6H_2_O and *n*-octadecane at 3500s timestamp.

**Fig. 16:**
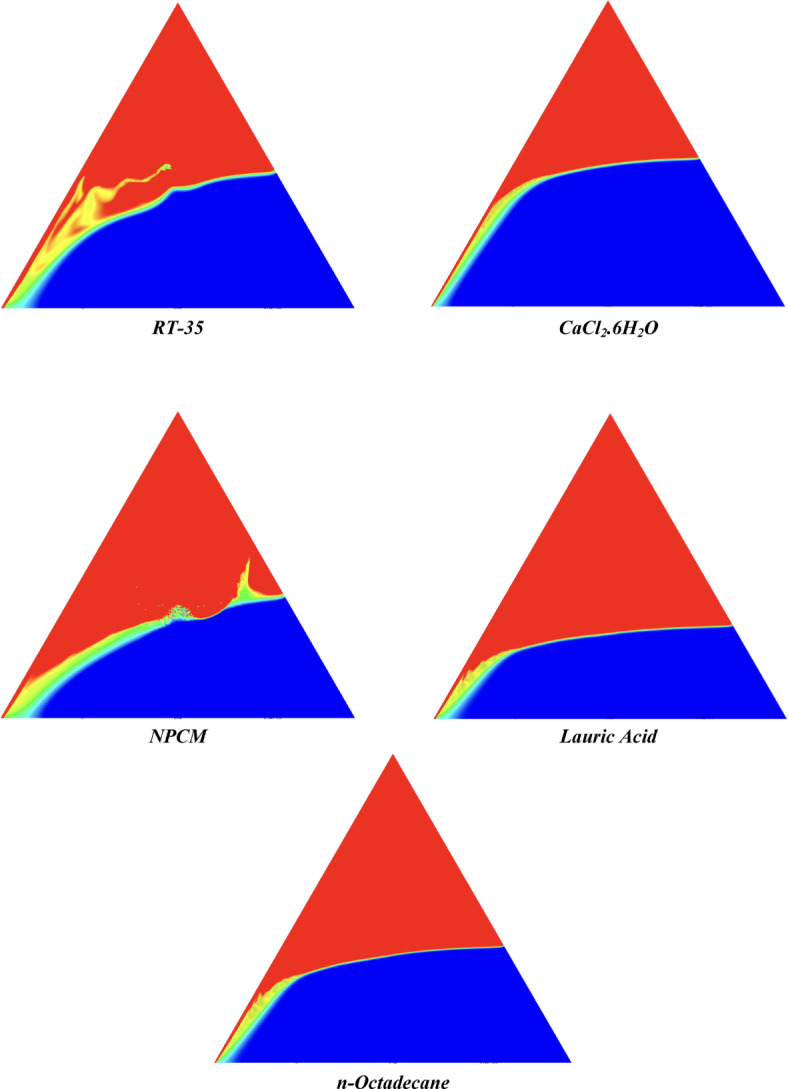
Melting regime of PCMs RT-35, Lauric Acid, NPCM, CaCl_2_·6H_2_O and *n*-octadecane at 4000s timestamp.

**Fig. 17:**
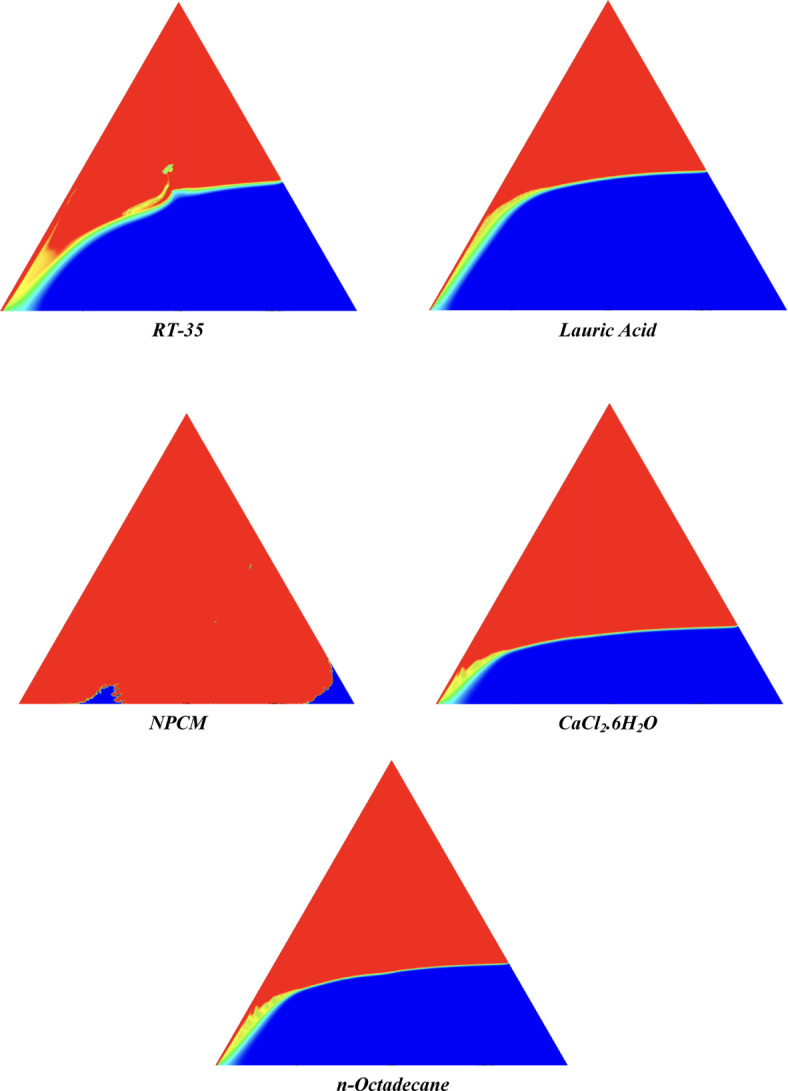
Melting regime of PCMs RT-35, Lauric Acid, NPCM, CaCl_2_·6H_2_O and *n*-octadecane at 4500s timestamp.

**Fig. 18:**
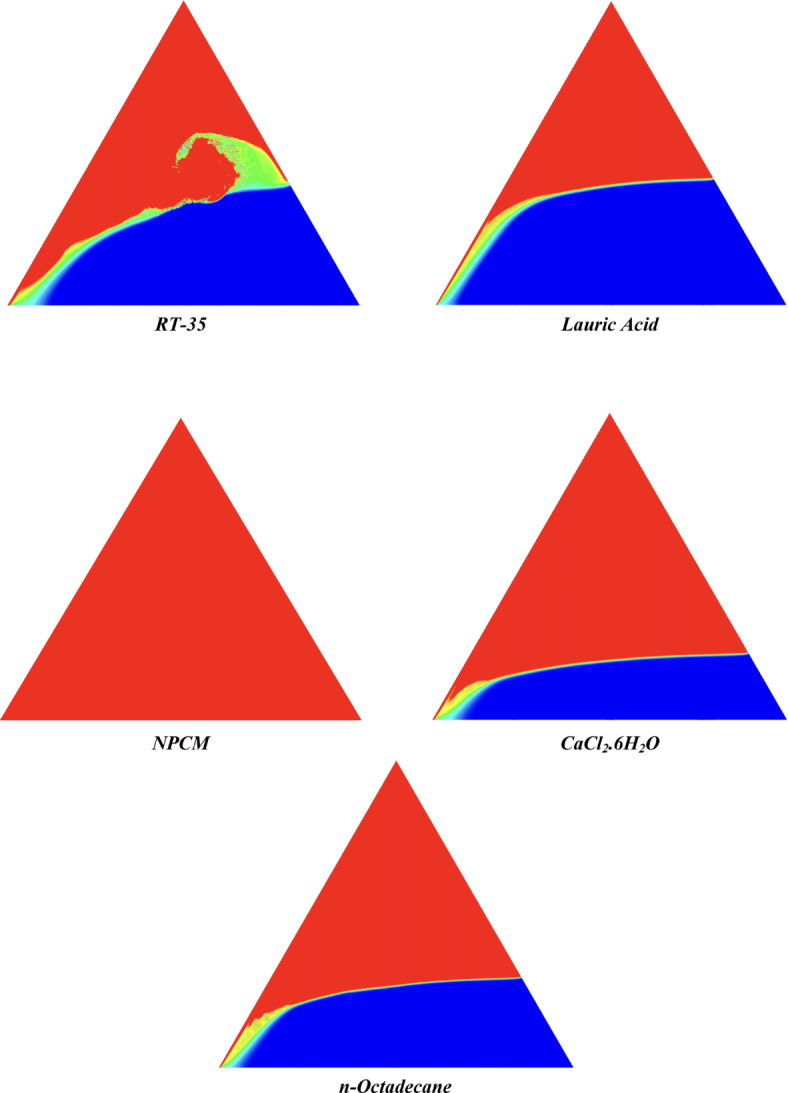
Melting regime of PCMs RT-35, Lauric Acid, NPCM, CaCl_2_·6H_2_O and *n*-octadecane at 5000s timestamp.

**Fig. 19:**
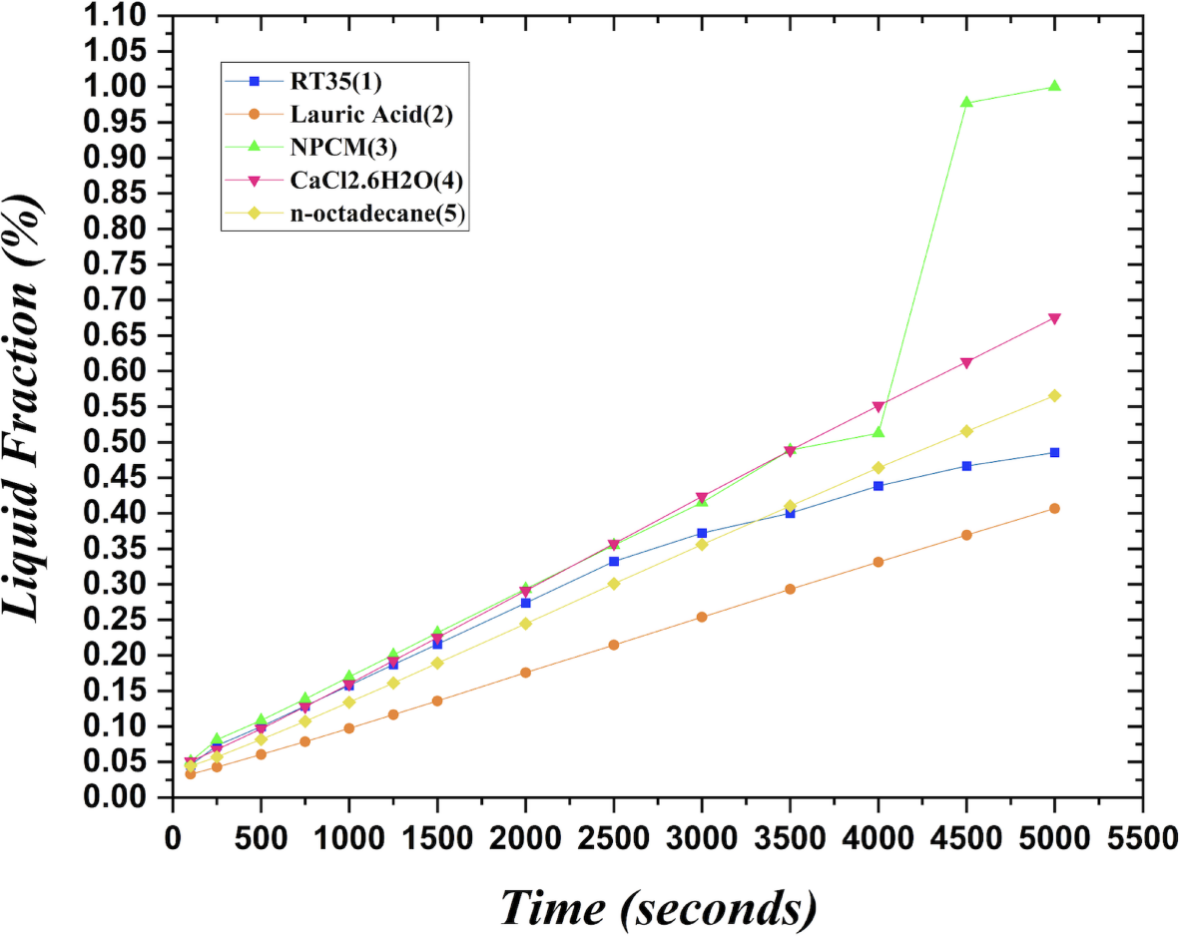
Comparison graph displaying the liquid fraction achieved at various timestamps as per numerical investigations for the various tested PCMs.

The melting regimes for each timestamp have been discussed in the following section. The results at 100 s as shown in Fig. 5, demonstrate that NPCM has a liquid fraction of 0.0511, which is higher than the liquid fractions of RT35, Lauric Acid, CaCl_2_·6H_2_O, and *n*-octadecane by approximately 10.35%, 35.57%, 0.46%, and 12.66% respectively. The formation of the mushy zone is very small and obscure at this timestamp. At 250 s as shown in Fig. 6, we observe that NPCM has a liquid fraction of 0.08156, still higher than the others by 10.41%, 47.55%, 17.28%, and 29.78% respectively. From this point onwards, the mushy zone becomes visible in all cases. The tip of the triangular geometry has clearly dissolved in all cases. At 500 s as shown in Fig. 7, NPCM continues to maintain the highest liquid fraction at 0.108, greater than the others by 8.02%, 44.24%, 10.81%, and 24.6% respectively. The mushy zone starts to thicken for CaCl_2_·6H_2_O as compared to the others. At 750 s as shown in Fig. 8, NPCM's liquid fraction remains higher than the rest at 0.139, exceeding the others by 7.194%, 43.31%, 7.914%, and 22.66% respectively. The melting of Lauric acid is the slowest up until this point. This could be because of its low thermal conductivity as shown in Table 1. At 1000 s as shown in Fig. 9, NPCM registers a liquid fraction of 0.17, surpassing the liquid fractions of the rest by 7.06%, 42.64%, 5.88%, and 21.18% respectively. At this juncture, a very small change in the liquid fraction was recorded for all the PCMs. At 1250 s as shown in Fig. 10, NPCM's liquid fraction is 0.2, still higher compared to the rest by 6.5%, 41.5%, 3.75%, and 19.4% respectively. A very little change can be observed by us however, the data goes to show that as the timestamp is increasing the liquid fraction is only going up. At 1500 s as shown in Fig. 11, NPCM's liquid fraction remains higher than the rest by 6.89%, 41.38%, 3.02%, and 18.53% respectively. The liquid fractions of all PCM have crossed the 15% mark which can be verified from Table 2; further, the liquid fractions of RT35, NPCM and CaCl_2_·6H_2_O have crossed the 20% mark which can be verified from Table 2. At 2000 s as shown in Fig. 12, NPCM's liquid fraction is still greater than the rest by 6.485%, 39.932%, 0.68%, and 16.38% respectively. It's worth noting that CaCl_2_·6H_2_O and NPCM have melted by almost a third of the original state. Moreover, the difference between the liquid fraction of the two is negligible which can be verified from Table 2. At 2500 s as shown in Fig. 13, CaCl_2_·6H_2_O has a higher liquid fraction than RT35, Lauric Acid, NPCM, and *n*-octadecane by approximately 7.00%, 39.776%, 0.56%, and 15.966% respectively, standing at roughly 0.357. Even though the difference in the liquid fraction is minute between CaCl_2_·6H_2_O and NPCM which can be verified from Table 2 however, it seems that a considerable portion of CaCl_2_·6H_2_O has melted away. At 3000 s as shown in Fig. 14, CaCl_2_·6H_2_O liquid fraction surpasses RT35, Lauric Acid, NPCM, and *n*-octadecane by approximately 12.19%, 40.094%, 2.051%, and 16.0377% respectively, with a value of roughly 0.424. The formation of mushy zones cannot be explained correctly as they are inexplicable however, in the case of RT35, we can see a small amount of turbulence which is believed to be part of the mushy zones. At 3500 s as shown in Fig. 15, NPCM achieves a liquid fraction of 0.4889, higher than the liquid fractions of RT35, Lauric Acid, CaCl_2_·6H_2_O and *n*-octadecane by approximately 18.18%, 40%, 0.04%, and 16.056% respectively. Uniform mushy zones with finger-like projections are seen in Lauric Acid, CaCl_2_·6H_2_O and *n*-octadecane. A small amount of turbulence is observed in the mushy zone of NPCM. The mushy zone of RT35 is a little spread out. At 4000 s as shown in Fig. 16, CaCl_2_·6H_2_O surpasses RT35, Lauric Acid, NPCM, and *n*-octadecane by approximately 20.475%, 39.86%, 7.04%, and 15.85% respectively, reaching a value of roughly 0.55. It is notable that NPCM and CaCl_2_·6H_2_O have both completed 50% of the melting process by this time. The mushy zone in RT35 becomes highly unstable and widely spread over. The turbulence increases even more in NPCM. Lauric acid finally completes a third of its melting which can be verified from Table 2. At 4500 s as shown in Fig. 17, NPCM is very close to complete melting, as it shows a high liquid fraction of 0.977 as shown in Table 2, higher than the liquid fractions of RT35, Lauric Acid, CaCl_2_·6H_2_O, and *n*-octadecane by approximately 52.24%, 62.23%, 37.257%, and 47.29% respectively. Notably, *n*-octadecane has also completed 50% of the melting process after this timestamp which can be verified from Table 2 which is greater than its organic counterpart, Lauric Acid, which has completed only 37% of its melting. NPCM melted completely sometime between 4500 and 5000 s, resulting in a 100% liquid fraction as shown in Fig. 18. Compared to RT35, Lauric Acid, CaCl_2_·6H_2_O, and *n*-octadecane, its liquid fraction is higher by 51.43%, 59.3175%, 32.45%, and 43.442% respectively. On comparing the two organic PCMs, Lauric Acid and *n*-octadecane, *n*-octadecane yields a much better result as it is able to attain a liquid fraction of approximately 0.57 and the latter only attains a liquid fraction of 0.4 which can be verified from Table 2. The turbulence formed in RT35 at the end of 5000 s is very random and the explanation for this could be the ambient temperature exceeding the melting temperature of the PCM which adds to the randomness of the mushy zone. The linearly varying thermal conductivity of the NPCM must have aided its melting process as the temperature kept rising, which can also explain the sudden shoot in the melting fraction post 4000 s. Undisputedly, the RT35 + Fe_3_O_4_ nanoparticle-enhanced NPCM gives the best result as it melted completely at the end of the chosen timestamp which can be verified by referring to Fig. 18 and Table 2. The next best inorganic alternative to NPCM is CaCl_2_·6H_2_O which achieved a maximum liquid fraction of 67.5% at the end of 5000 s which can be verified by referring to Fig. 18, and Table 2. Similarly, RT35 and Lauric acid achieved a maximum liquid fraction of 48.57% and 40.625% respectively at the end of 5000 s which can be verified by referring to Fig. 18, and Table 2. From our results, the best organic alternative to NPCM is *n*-octadecane, which achieved a maximum liquid fraction of 56.58% at the end of the final timestamp which can be verified by referring to Fig. 18 and Table 2. Throughout the melting regime, the liquid fraction of RT35 increased steadily. A very small curve can be seen in the graph as shown in Fig. 19 post 1500 s perhaps because of RT35 attainting its melting temperature of 308K between 1500 and 2000s as mentioned in Table 2. Lauric acid is able to maintain a perfectly linear relation between the attained liquid fraction and the succeeding timestamp as shown in Fig. 19. This could be because of its low thermal conductivity which aids in lesser turbulence. In the case of NPCM, as shown in Fig. 19, the trend stays more or less linear till 4000 s, however, post 4000 s, a sudden increase in the melting fraction is observed. This can be explained using two factors. Firstly, after 2000s NPCM crosses its melting temperature as mentioned in Tables 1 and 2, making the melting process unsteady. Secondly, of all the selected PCMs only NPCM has a linearly varying thermal conductivity due to the incorporation of the Fe_3_O_4_ nanoparticles which can be seen in Table 1. This surely aids in its melting process and ensures greater facilitation of heat transfer. For CaCl_2_·6H_2_O, the change in the liquid fraction is steady and directly proportional to the timestamp as shown in Fig. 19. This steady relationship can be explained with the help of the thermophysical properties of CaCl_2_·6H_2_O. Since the melting temperature of CaCl_2_·6H_2_O is low which is attained at 500 s as mentioned in Table 2 and it does not have a varying thermal conductivity as shown in Table 1, the trend remains steady. For *n*-octadecane a similar explanation for the linearly varying curve as shown in Fig. 19 can be given as that of CaCl_2_·6H_2_O however, it is interesting to note that despite having the same melting temperature the extent of liquid fraction in the case of *n*-octadecane and CaCl_2_·6H_2_O varies greatly majorly due to the difference in the appreciable difference in their thermal conductivities as shown in Table 1. In our final combined study as shown in the comparison graph in Fig. 19, we can observe that throughout the chosen time period, NPCM and CaCl_2_·6H_2_O have a higher liquid fraction than RT35, Lauric Acid and *n*-octadecane. NPCM prevails as the most suitable PCM post approximately 4300 s. Lauric Acid remains the least melted PCM and its graph hence remains at the bottom of the comparison graph. Between RT35 and *n*-octadecane we observe that right sometime before 3250 s RT35 had a greater liquid fraction however post the 3250 s, *n*-octadecane had a higher liquid fraction.

**Table 1:** Thermophysical properties of selected PCMs^3,7,22,27^.

PCM	*T*_*m*_ (K)	*C*_*p*_ (J/kg-K)	*v* (m^2^/s)	*H*_*f*_ (kJ/kg)	*ρ* (kg/m^3^)	*K* (W/m K)	*β* (1/K)
RT35	308	2000	0.023	160	770	0.2	0.00061
Lauric acid	316	2110	0.006	187.2	880	0.15	0.001
NPCM	311	1838.9	0.02419	160	858.6	0.258 (298 K) 0.295 (333 K)	0.00053754
CaCl_2_·6H_2_O	301	2060	0.01	170	1538	0.51	0.0005
*n*-octadecane	301	2150	0.0085	189	814	0.2	0.00085

**Table 2:** Melting data as per numerical study for all selected PCMs at different time stamps using a mesh size of 0.1 mm.

Timestamp (s)	Liquid fraction of PCMs				
	RT-35	Lauric acid	NPCM	CaCl_2_·6H_2_O	*n*-octadecane
100	0.04583549	0.03293951	0.0511273	0.05089185	0.04465261
250	0.07318552	0.04283892	0.08155957	0.06748356	0.05730959
500	0.09982525	0.06054725	0.1085378	0.09677459	0.08182622
750	0.1288068	0.07878566	0.1389239	0.1279173	0.1074967
1000	0.1578867	0.09747964	0.1699684	0.1598267	0.1341083
1250	0.1869599	0.1167148	0.2008986	0.1925408	0.1612212
1500	0.2160495	0.1361217	0.2317391	0.2249286	0.1893483
2000	0.274049	0.1757918	0.2933927	0.2911213	0.2447392
2500	0.3323335	0.2148733	0.3550859	0.357432	0.3009894
3000	0.3723779	0.2538945	0.4153693	0.423773	0.3559331
3500	0.4003975	0.2932944	0.4889263	0.4887682	0.4103749
4000	0.4385315	0.3316286	0.5125944	0.551416	0.4640587
4500	0.4665992	0.3693383	0.9772138	0.613106	0.5153408
5000	0.4856957	0.406825	1.0000000	0.6755059	0.5655854

## Conclusion

In our presented work, we have numerically analyzed the melting of five different PCM’s to utilize them to enhance the efficiency of single-slope solar still. The study was done with the motivation of taking a step closer towards integrating the application of PCM in desalination. The following conclusions are drawn from the numerical study:

- The comparative analysis of the melting behavior of the different PCMs revealed that NPCM outperformed the other candidates in terms of faster and more efficient melting. Its superior thermal characteristics make it an ideal choice for integration into a PCM-embedded single-slope solar still. By enhancing the heat absorption and release processes, NPCM can significantly increase the desalination efficiency, making it a prime material for sustainable and effective thermal energy storage in solar still applications.
- The incorporation of Fe_3_O_4_ nanoparticles into RT35 led to a noticeable improvement in its thermal properties, particularly in terms of faster heat transfer and reduced melting time. This hybrid PCM-nanofluid combination demonstrates the potential of using nanoparticles to enhance the thermal conductivity of conventional PCMs. As a result, the solar still can maintain higher temperatures for longer periods, increasing the overall water production. The study underscores the importance of exploring nanoparticle-enhanced PCMs for optimizing solar thermal applications.
- While NPCM showed the best overall performance, *n*-octadecane, an organic PCM, also demonstrated considerable potential in enhancing solar still productivity. Its environmental friendliness, wide availability, and cost-effectiveness make it an attractive alternative for large-scale desalination projects. Despite being third in the ranking, its combination of sustainability and adequate performance highlights its role as a viable, eco-friendly option for promoting renewable energy solutions in desalination processes.
- The use of a triangular cross-section in the solar still design played a crucial role in reducing the PCM melting time by improving heat transfer efficiency. This geometric configuration has a higher conduction shape factor, which minimizes thermal resistance and promotes faster heat diffusion throughout the PCM. The results suggest that optimizing the geometry of solar stills can significantly impact their thermal performance, further enhancing desalination rates. The adoption of such innovative design features can lead to more energy-efficient and productive solar stills.

## Data Availability

The datasets used and/or analysed during the current study available from the corresponding author on reasonable request.

## Abbreviations

PCM: Phase change material
NPCM: Nanoparticle phase change material (RT35 + Fe_3_O_4_ nanoparticle)

## List of symbols

*Β*: Coefficient of thermal expansion (1/K)
*K*: Thermal conductivity (W/m K)
*v*: Kinematic viscosity (kg/m s)
*ρ*: Density (kg/m^3^)
*t*: Time (s)
*T*: Temperature (°C)
*T*_*m*_: Melting temperature (°C)
*C*_*p*_: Specific heat capacity (J/kg K)
*H*_*f*_: Heat of fusion (kJ/kg)
*u*: Velocity in the radial direction
*w*: Velocity in the axial direction
*g*: Gravitational acceleration

## References

[1] Moreno, S., Álvarez, C., Hinojosa, J. F. & Maytorena, V. M. Numerical analysis of a solar still with phase change material under the basin. *J. Energy Storage* **55**, 105427 (2022).

[2] Manoj Kumar, P. *et al.* Performance study on solar still using nano disbanded phase change material (NDPCM). *Mater. Today Proc.* **62**, 1894–1897 (2022).

[3] Hussain, F. *et al.* Transient solidification and melting numerical simulation of lauric acid PCM filled stepped solar still basin used in water desalination process. *Case Stud. Therm. Eng.* **49** (2023).

[4] Gugulothu, R., Somanchi, N. S., Devi, R. S. R. & Banoth, H. B. Experimental investigations on performance evaluation of a single basin solar still using different energy absorbing materials. *Aquat. Procedia* **4**, 1483–1491 (2015).

[5] Khandagre, M., Gupta, B., Bhalavi, J. & Baredar, P. Magnesium sulfate heptahydrate as phase change material in double slope solar still. *J. Therm. Eng.* **7** (2021).

[6] Al-Hamadani, A. A. F. & Shukla, S. K. Modelling of solar distillation system with phase change material storage medium. *Therm. Sci.* **18**, 347–362 (2014).

[7] Maduabuchi, C. C. & Mgbemene, C. A. Numerical study of a phase change material integrated solar thermoelectric generator. *J. Electron. Mater.* **49**, 5917–5936 (2020).

[8] Abdullah, A. S., Essa, F. A., Bacha, H. Ben & Omara, Z. M. Improving the trays solar still performance using reflectors and phase change material with nanoparticles. *J. Energy Storage* **31** (2020).

[9] Seifi, J., Samimi-Akhijahani, H. & Salami, P. Investigating the thermo-economic performance and modeling of a parabolic solar collector with phase change material in the receiver tube in a solar desalination. *Desalination* **572** (2024).

[10] Santhosh, S., Satish, M., Madhavan, A. A. & Yadav, A. Solar still with integrated solar heater and nanoparticle-enhanced energy storage material. *J. Electron. Mater.* **53**, 1956–1963 (2024).

[11] Raju, G., Dillibabu, S. P., Tonk, A. & Kumar Shanmugam, S. A comparative study for enhancingsolar still performance and efficiencythrough PCM-integrated fin design. *Therm. Sci.* **27**, 4841–4850 (2023).

[12] Manoj Kumar, P. et al. Experimental investigations on the performance of a single slope solar still with thermal energy storage. *Mater. Today Proc.*10.1016/j.matpr.2022.12.221 (2022).

[13] Dhivagar, R., Mohanraj, M., Hidouri, K. & Midhun, M. CFD modeling of a gravel coarse aggregate sensible heat storage assisted single slope solar still. *Desalin. Water Treat.* **210**, 54–69 (2021).

[14] El-Sebaey, M. S., Ellman, A., Hegazy, A. & Ghonim, T. Experimental analysis and CFD modeling for conventional basin-type solar still. *Energies (Basel)* **13** (2020).

[15] Kabeel, A. E., El-Samadony, Y. A. F. & El-Maghlany, W. M. Comparative study on the solar still performance utilizing different PCM. *Desalination* **432**, 89–96 (2018).

[16] Omara, A. A. M., Abuelnuor, A. A. A., Mohammed, H. A. & Khiadani, M. Phase change materials (PCMs) for improving solar still productivity: A review. *J. Therm. Anal. Calorim.* **139**, 1585–1617 (2020). 10.1007/s10973-019-08645-3

[17] Ketut, C. N. I., Yusvardi, Y. & Muhamad, K. F. Myristic acid as phase change material (PCM) for increased productivity of solar distillation plant. *Int. Rev. Appl. Sci. Eng.* **11**, 226–231 (2020). 10.1556/1848.2020.00077

[18] Li, J. *et al.* Thermal performance analysis of composite phase change material of myristic acid-expanded graphite in spherical thermal energy storage unit. *Energies (Basel)* **16** (2023).

[19] Faraj, K., Khaled, M., Faraj, J., Hachem, F. & Castelain, C. *Phase Change Material Thermal Energy Storage Systems for Cooling Applications in Buildings: A Review*. https://www.sciencedirect.com/science/article/pii/S1364032119307877.

[20] Khairat Dawood, M. M. *et al.* Increasing the freshwater productivity of a solar still loaded with CuO nanofluids using vibration motion and cover cooling techniques. *Int. J. Energy Res.* **45**, 9099–9115 (2021).

[21] Dawood, M. M. K. et al. 3E enhancement of freshwater productivity of solar still with heater, vibration, and cover cooling. *Environ. Sci. Pollut. Res.* **29**, 65787–65805 (2022). 10.1007/s11356-022-20340-9 PMC9481515 35499732

[22] Pan, C. et al. Experimental, numerical and analytic study of unconstrained melting in a vertical cylinder with a focus on mushy region effects. *Int. J. Heat Mass Transf.* **124**, 1015–1024 (2018).

[23] Chelliah, A., Saboor, S., Ghosh, A. & Kontoleon, K. J. Thermal behaviour analysis and cost-saving opportunities of PCM-integrated terracotta brick buildings. *Adv. Civ. Eng.* (2021).

[24] Hedwig, M., Schroeder, P. W. N., Hedwig, M. & Schroeder, P. W. A grad-div stabilized discontinuous galerkin based thermal optimization of sorption processes via phase change materials.

[25] Wong-Pinto, L. S., Milian, Y. & Ushak, S. Progress on use of nanoparticles in salt hydrates as phase change materials. *Renew. Sustain. Energy Rev.* (2020)*.*10.1016/j.rser.2020.109727

[26] Soodmand, A. M., Nejatbakhsh, S., Pourpasha, H., Aghdasinia, H. & Heris, S. Z. Simulation of melting and solidification process of polyethylene glycol 1500 as a PCM in rectangular, triangular, and cylindrical enclosures. *Alex. Eng. J.* **61**, 8431–8456 (2022).

[27] Ebadi, S., Al-Jethelah, M., Tasnim, S. H. & Mahmud, S. An investigation of the melting process of RT-35 filled circular thermal energy storage system. *Open Phys.* **16**, 574–580 (2018).

[28] Simeza, L. M. & Yovanovich, M. M. Shape factors for hollow prismatic cylinders bounded by isothermal inner circles and outer regular polygons. *Int. J. Heat Mass Transf.* **30**, 812–816 (1987).

[29] Ebadi, S., Tasnim, S. H., Aliabadi, A. A. & Mahmud, S. Melting of nano-PCM inside a cylindrical thermal energy storage system: Numerical study with experimental verification. *Energy Convers. Manag.* **166**, 241–259 (2018).

